# Community benchmarking of generative models for privacy-preserving synthetic bulk RNA-seq data generation

**DOI:** 10.1186/s13059-026-04326-1

**Published:** 2026-11-09

**Authors:** Hakime Öztürk, Tejumade Afonja, Joonas Jälkö, Ruta Binkyte, Pablo Rodriguez-Mier, Sebastian Lobentanzer, Andrew Wicks, Jules Kreuer, Sofiane Ouaari, Nico Pfeifer, Shane Menzies, Sikha Pentyala, Daniil Filienko, Steven Golob, Patrick McKeever, Jineta Banerjee, Luca Foschini, Martine De Cock, Julio Saez-Rodriguez, Mario Fritz, Oliver Stegle, Antti Honkela

**Affiliations:** 1 European Molecular Biology Laboratory (EMBL), Genome Biology Unit, Heidelberg, Germany; 2 Division of Computational Genomics and Systems Genetics, German Cancer Research Center (DKFZ), Heidelberg, Germany; 3 CISPA Helmholtz Center for Information Security, Saarbrücken, Germany; 4 University of Helsinki, Helsinki, Finland; 5 Faculty of Medicine, Institute for Computational Biomedicine, Heidelberg University, Heidelberg, Germany; 6 Helmholtz Munich, Munich, Germany; 7 Division of Tumorigenesis and Molecular Cancer Prevention, German Cancer Research Center (DKFZ), Heidelberg, Germany; 8 DKFZ Hector Cancer Institute at the University Medical Center Mannheim, Mannheim, Germany; 9 Eberhard Karls Universität Tübingen, Tübingen, Germany; 10 University of Washington Tacoma, Washington, USA; 11 Sage Bionetworks, Seattle, USA; 12 Ghent University, Ghent, Belgium; 13 European Molecular Biology Laboratory, European Bioinformatics Institute (EMBL-EBI), Hinxton, UK; 14 Heidelberg University Hospital, Heidelberg, Germany

**Keywords:** Synthetic data generation, Private data generation, RNA-seq, Generative models, Membership inference attack, Reproducibility, Evaluation metrics and trade-offs, Biological plausibility

## Abstract

**Background:**

The synthesis of anonymized data derived from real-world cohorts offers a promising strategy for regulatory-compliant and privacy-preserving biological data sharing, potentially facilitating model development that can improve predictive performance. However, the extent to which generative models can preserve biological signals while remaining resilient to adversarial privacy attacks in high-dimensional omics contexts remains underexplored. To address this gap, the CAMDA 2025 Health Privacy Challenge launched a community-driven effort to evaluate synthetic and privacy-preserving data generation for bulk RNA-seq cohorts.

**Results:**

Building on this initiative, we systematically benchmark 11 generative methods across two cancer cohorts (~ 1,000 and ~ 5,000 patients) over 978 landmark genes. Methods are evaluated across distributional fidelity, downstream utility, biological plausibility and empirical privacy risk, with emphasis on trade-offs between vulnerability to membership inference attacks and other evaluation dimensions. Expressive deep generative models achieve strong predictive utility and differential expression recovery, but are often more vulnerable to membership inference risk under the evaluated threat model. Differentially private methods improve resistance to attacks at the cost of reduced utility, while simpler statistical approaches offer competitive utility with moderate privacy risk and fast training.

**Conclusions:**

Synthetic bulk RNA-seq quality is inherently multi-dimensional and shaped by trade-offs between utility, biological preservation and privacy. Our results indicate that model architecture drives distinct trade-offs across these axes, suggesting that model choice should align with dataset characteristics, intended downstream use and privacy requirements. Privacy risk should also be assessed using multiple complementary attack methods and, where possible, mitigated through formal differential privacy protection.

**Supplementary Information:**

The online version contains supplementary material available at https://doi.org/10.1186/s13059-026-04326-1.

## Background

The unprecedented availability of health data facilitates understanding and predicting disease and patient trajectories. Machine and deep learning-based methods learn latent representations from large amounts of datasets, potentially improving generalization to unknown and rare cases. However, the improved performance of these methods often depends on large parameter spaces that are prone to memorisation of data points, posing challenges for patient privacy. While on one hand, the benefit of sharing and utilizing large scale health care data promises increased utility, it is also crucial to ensure patient privacy is not disclosed. Provable privacy preservation can be implemented through differential privacy (DP) [1, 2]. Federated learning (FL) [3] and synthetic data generation [4–6] can provide some protection alone, or be combined with DP.

Synthetic data generation enables privacy preservation through generating data points that approximate the distribution of the real data without exposing individual records. This also creates opportunities for data augmentation and building fair models [7–9]. However, synthetic data are not unconditionally private and can still be vulnerable to privacy attacks [10–12], motivating rigorous empirical evaluation of privacy risk alongside utility. The utility and privacy of synthetic data generated for Electronic Health Records [13–16], genomics [17–21] and gene expression (RNA-seq) [22–24] has been investigated through different evaluation metrics. The “Hide and Seek Privacy Challenge”, organized as a part of NeurIPS 2020, provided a platform for teams to generate synthetic longitudinal intensive care data and to launch privacy attacks on the generated data to compete against each other [25]. The results of the competition highlighted the complexities of balancing utility and privacy in synthetic biological datasets, emphasizing the need for developing robust privacy-preserving generative methods and for reliable evaluation metrics.

Although recent studies have been examining trade-offs among utility, fidelity, and privacy metrics [22, 26–29], the way these metrics interact across diverse generative-model families remains underexplored, particularly for gene-expression datasets, which are high dimensional and exhibit biologically structured patterns, including gene–gene co-expression dependencies and condition-specific differential expression. Gene expression profiles carry sufficient signal to re-identify individuals through genotype-expression linking attacks [30–32]. Expression variation encodes population structure, disease state, and treatment response; therefore, sharing individual-level profiles carries a privacy risk. While bulk RNA-seq matrices from cohorts such as The Cancer Genome Atlas (TCGA) [33] are publicly accessible, many gene expression datasets (e.g. clinical or institutional cohorts) cannot be openly shared, in part because of such re-identification risks alongside broader consent and regulatory constraints. Synthetic data generation offers a route to release distributionally and biologically faithful surrogates while reducing disclosure risk. Bulk RNA-seq therefore represents a well-motivated and practically relevant testbed to study trade-offs between biological signal preservation, utility and privacy in synthetic data. It is widely available across cohorts, supports different downstream analyses, and is commonly represented in tabular format, lowering the barrier to evaluation under realistic data-sharing constraints. We use TCGA as an accessible benchmark, while the underlying motivation applies to many molecular datasets that cannot be shared openly.

The Health Privacy Challenge (https://benchmarks.elsa-ai.eu/?ch=4 [34]) organized in conjunction with the Critical Assessment of Massive Data Analysis (CAMDA) 2025, was designed to collectively explore privacy-preserving synthetic data generation in the context of gene expression datasets. The first track of the challenge focused on private synthetic bulk RNA-seq generation for two TCGA datasets, where “blue” teams generated biologically meaningful and private synthetic datasets, and “red” teams investigated the vulnerabilities of these generated datasets at the level of membership inference attack (MIA) [35]. In MIA, an adversary aims to determine the presence of an individual’s data in the training set of a generative model. Although membership inference attacks can pose a privacy risk in regulated or stigmatized sensitive datasets, they often reveal limited information but are widely used as a foundational primitive to evaluate vulnerability to more serious attacks such as attribute inference and data reconstruction [36]. We adopt MIA as our privacy axis because it directly tests the central privacy claim of synthetic data, that individual training records cannot be identified from release, and provides a standard and quantifiable indicator of privacy risk. The challenge setting, therefore, provides a controlled environment in which utility, fidelity, biological signal preservation, and privacy risk can be evaluated under comparable conditions.

In this work, we build on the first track’s “Blue” team task of the Health Privacy Challenge 2025 which addresses private synthetic bulk RNA-seq data generation and received the majority of submissions (Additional file 1: Challenge description). Extending prior evaluations focused primarily on differentially private models [22, 23], we benchmark a broader range of generative approaches and assess their performance across complementary dimensions. We harmonize community submissions with suggested baseline and post-challenge methods to benchmark models for private synthetic bulk gene-expression generation. Rather than emphasizing model ranking or competitive outcomes, we analyze how different classes of metrics such as utility, global fidelity, biological preservation, and privacy relate to one another, where they diverge, and how sensitive their conclusions are to model architecture, dataset characteristics, threat models and differential privacy constraints. This enables explicit quantification of trade-offs and provides actionable guidance for model selection in realistic cohort settings. Overall, this study, (i) provides a summary of the current landscape of approaches, (ii) establishes a reference point for future research and community challenges, and (iii) highlights the need for multi-dimensional evaluation practices for synthetic transcriptomic datasets.

## Results

**Fig. 1:**
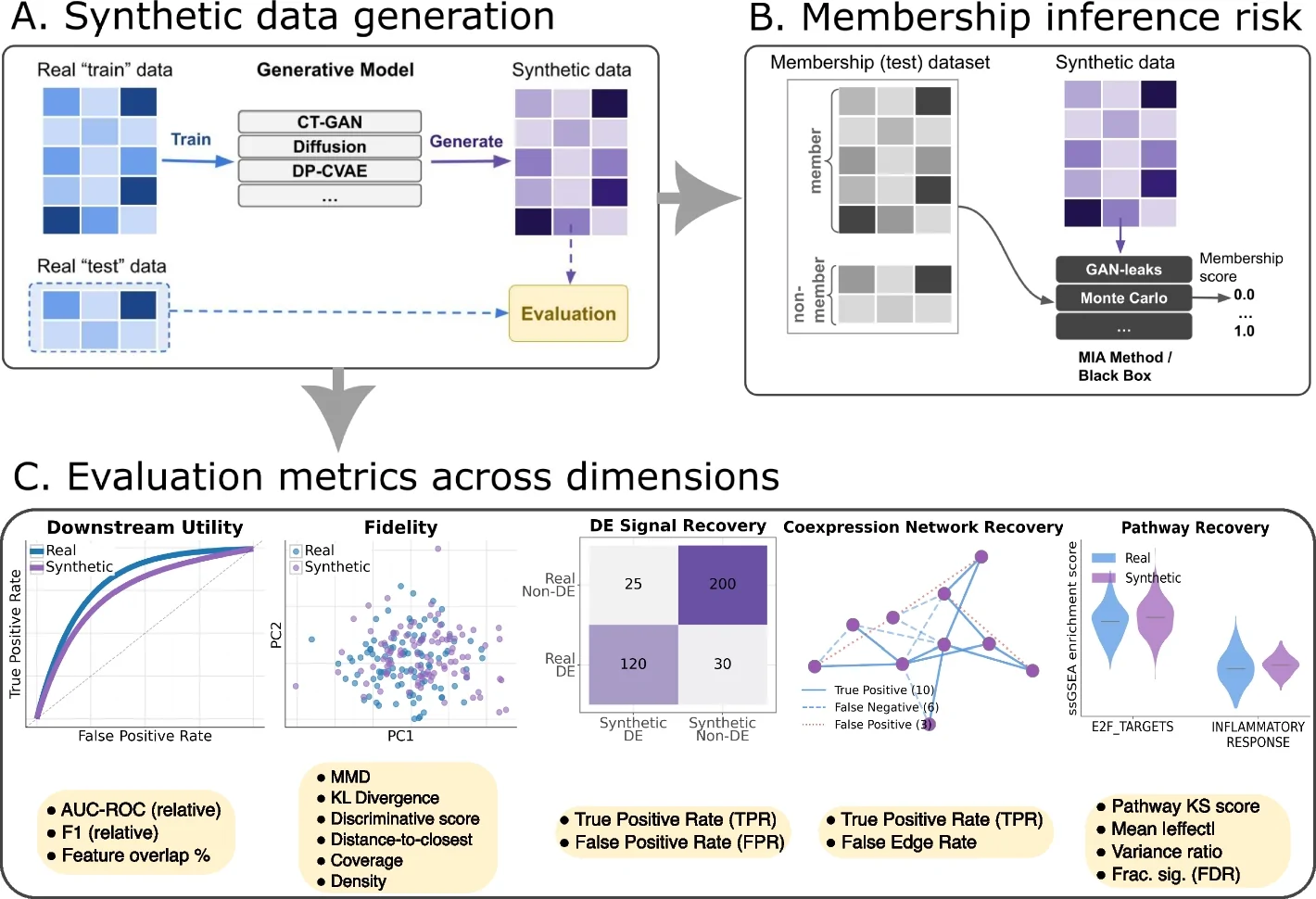
Benchmarking framework for evaluating generative models on synthetic bulk RNA-seq data generation. **A** Synthetic data generation workflow. Real bulk RNA-seq data are partitioned into training and test sets. Generative models (including differentially private methods, denoted by DP) are trained exclusively on the training data to generate synthetic samples. The test set is held out for evaluation purposes and not used during model training or data generation. **B** Privacy risk assessment via membership inference attacks (MIA).Real data is labeled as members (samples present in the training set) and non-members (held-out (test) samples) to generate a membership (query) test dataset. Both membership test dataset and synthetic data are input to black-box MIA methods (e.g. GAN-leaks [37] etc.) to predict membership scores, which quantify the risk of identifying whether specific samples were used in model training. MIA performance is quantified by AUC-ROC and true positive rate (TPR) fixed at specific false positive rate (FPR) value (i.e. TPR@FPR = 0.1) to assess potential disclosure of training samples. **C** Evaluation metrics across dimensions. Models are evaluated across complementary dimensions: (i) Distributional Fidelity: statistical similarity between real and synthetic distributions assessed via maximum mean discrepancy (MMD), Kullback–Leibler (KL) divergence, distance-to-closest real record, and discriminator-based metrics; (ii) Downstream Utility: preservation of predictive performance measured by relative AUC-ROC, F1 score, and overlap of important features when training classifiers on synthetic versus real data; (iii) Biological Plausibility: recovery of biologically meaningful patterns including differential expression (DE) (true positives, false negatives, false positives etc.), co-expression network edge recovery (true positives in blue, false negatives in dashed blue, false positives in dotted red) and pathway score distributions, illustrating how synthetic data (purple) may match, shift relative to, or under-disperse compared with real data (blue)

We generated synthetic bulk RNA-seq data based on two patient-level cohorts, TCGA-BRCA (breast cancer dataset with molecular subtypes) and TCGA-COMBINED (multi-tissue dataset), using 8 distinct generative architectures, three of which evaluated with and without a privacy mechanism (11 methods in total). We assessed the quality of the generated synthetic datasets across four key dimensions: distributional fidelity, downstream utility, biological plausibility, and privacy (Fig. 1). The complete list of all metrics considered in this benchmarking study is provided in Additional file 2: Table S1.

The formally differentially private (DP) models DP-CVAE and DP-PGM were evaluated at a privacy budget of ∈=4, a relatively weak formal guarantee, adopted as a pragmatic operating point that retains sufficient utility. The results across $\in =\mathrm{1,4},\mathrm{10,50}$ are provided as an ablation (Additional file 3: Fig. S1). DP-CTGAN could only be evaluated at a looser budget $\left(\in =50\right)$, as its preprocessing consumes a part of the privacy budget under composition, and therefore provides substantially weaker privacy guarantees. Privatized-NMF (P-NMF) applies calibrated noise to the released per-cluster gene means; however, the synthetic data it produces do not carry a formal differential-privacy guarantee.

### Distributional fidelity and separability of synthetic bulk RNA-seq across models

Fidelity measures how closely the synthetic data reproduce the global and local structure of the real data. We evaluated distributional fidelity using complementary global (Maximum Mean Discrepancy (MMD) and Kullback–Leibler (KL) divergence), local (Distance-to-closest record), manifold-based (density and coverage [38]) and discriminative score (classifier-based)-based metrics, computed against both the real train and the real held-out test data (Methods). We report test-set-referenced MMD, KL, density, and coverage to emphasize generalization of synthetic data to held-out real samples. Training-referenced counterparts were generally correlated with their test-based versions, strongly for MMD, KL, and coverage, and positively though more weakly for density (Additional file 3: Figs. S2-S3), indicating that the choice of reference (i.e. train or test) does not materially alter the relative ranking of generators. Table 1 summarizes these fidelity metrics across generative models.

**Table 1:** Fidelity metrics across TCGA-BRCA and TCGA-COMBINED datasets. We report the mean of each metric across five folds, with standard deviation in parentheses. Directionality arrows (↓ ↑) indicate the direction of better performance. Relative (rel.) metrics report the performance of the synthetic data relative to real data and the directionality indicator reflects whether exceeding real-data performance is beneficial (↑) or whether a match to real data (~ 1) is the target. (~ 1) indicates a value near 1 is most faithful; larger or smaller values indicate over- or under-representation relative to real data. For each metric, the best-performing model (and any model within one standard deviation of it) is shown in **bold,** and the next-best is underlined. Metrics whose optimal value is a target (~ 1) are not ranked. Bolding and underlining are determined within each dataset independently; the two datasets are shown together for compactness and are not directly compared. (Disc. score: discriminative score)

	**MVN**	**CVAE**	**DP-CVAE ε = 4**	**CTGAN**	**DP-CTGAN ε = 50**	**EMB-Diffusion**	**NMF**	**P-NMF ε = 4**	**DP-PGM ε = 4**	**WGAN-GP**	**CVAE-GMM**
**TCGA-BRCA**											
**MMD (test) ↓**	0.019 (0.001)	0.024 (0.004)	0.266 (0.003)	0.094 (0.016)	1.120 (0.024)	0.014 (0.003)	**0.003 (0.000)**	0.291 (0.003)	0.013 (0.001)	0.033 (0.002)	0.054 (0.006)
**KL (test) ↓**	**0.215 (0.006)**	0.650 (0.043)	0.930 (0.008)	5.762 (0.071)	19.857 (0.189)	0.407 (0.030)	0.286 (0.013)	2.930 (0.059)	18.500 (0.059)	0.831 (0.044)	1.397 (0.095)
**Discr. score ↓**	**0.528 (0.021)**	0.788 (0.012)	1.000 (0.000)	0.996 (0.003)	1.000 (0.000)	0.633 (0.025)	0.683 (0.011)	1.000 (0.000)	0.961 (0.009)	0.705 (0.025)	0.694 (0.029)
**DCR (rel.) ~ 1**	1.187 (0.015)	0.642 (0.029)	3.449 (0.046)	0.819 (0.016)	3.746 (0.073)	0.894 (0.034)	1.154 (0.011)	11.690 (0.246)	1.144 (0.013)	0.805 (0.015)	0.698 (0.009)
**Density (test) ~ 1**	0.062 (0.018)	3.855 (0.330)	0.000 (0.000)	9.321 (0.504)	0.000 (0.000)	5.434 (0.347)	0.001 (0.001)	0.000 (0.000)	0.004 (0.004)	8.553 (0.823)	12.092 (0.197)
**Coverage (test) ↑**	0.352 (0.040)	0.996 (0.004)	0.000 (0.000)	0.985 (0.014)	0.000 (0.000)	**1.000 (0.000)**	0.007 (0.005)	0.000 (0.000)	0.013 (0.006)	0.997 (0.004)	**1.000 (0.000)**
**TCGA-COMBINED**											
	**MVN**	**CVAE**	**DP-CVAE ε = 4**	**CTGAN**	**DP-CTGAN ε = 50**	**EMB-Diffusion**	**NMF**	**P-NMF ε = 4**	**DP-PGM ε = 4**	**WGAN-GP**	**CVAE-GMM**
**MMD (test) ↓**	0.009 (0.000)	0.026 (0.002)	0.115 (0.003)	0.087 (0.007)	0.906 (0.060)	0.006 (0.000)	**0.002 (0.000)**	0.144 (0.002)	0.008 (0.000)	0.014 (0.003)	0.026 (0.005)
**KL (test) ↓**	**0.074 (0.001)**	0.344 (0.029)	0.409 (0.022)	5.531 (0.036)	19.045 (0.393)	0.100 (0.003)	0.077 (0.005)	0.718 (0.026)	17.644 (0.049)	0.159 (0.021)	0.538 (0.126)
**Discr. score ↓**	**0.578 (0.011)**	0.788 (0.009)	1.000 (0.000)	0.996 (0.001)	1.000 (0.000)	0.723 (0.021)	0.641 (0.009)	1.000 (0.000)	0.757 (0.009)	0.953 (0.008)	0.790 (0.019)
**DCR (rel.) ~ 1**	1.241 (0.005)	0.711 (0.011)	2.898 (0.086)	1.131 (0.013)	4.471 (0.060)	0.950 (0.022)	1.235 (0.012)	3.580 (0.029)	1.297 (0.008)	0.865 (0.019)	0.695 (0.013)
**Density (test) ~ 1**	0.059 (0.007)	7.363 (0.510)	0.000 (0.000)	1.579 (0.157)	0.000 (0.000)	2.250 (0.262)	0.001 (0.001)	0.000 (0.000)	0.000 (0.000)	6.902 (0.398)	9.755 (0.640)
**Coverage (test) ↑**	0.283 (0.014)	1.000 (0.001)	0.000 (0.000)	0.368 (0.012)	0.000 (0.000)	0.996 (0.002)	0.005 (0.004)	0.000 (0.000)	0.000 (0.001)	0.997 (0.003)	**1.000 (0.000)**

Across both datasets, most non-private methods exhibit similarly low MMD scores with the addition of the Differentially Private (DP) model DP-PGM. However, differences emerge in KL divergence metric. Statistical methods, including the Multivariate Normal (MVN) and Non-negative Matrix Factorization (NMF), achieve comparatively lower KL divergence, followed by the Embedded Diffusion model on both datasets. DP-PGM $\left(\in =4\right)$ displays a surprisingly high KL divergence despite having a relatively low MMD score. This might indicate that, although synthetic data matches multivariate structure of real samples under RBF kernel (low MMD), it might fail to reproduce gene-wise marginal distributions (high KL). As a result, DP-PGM generates samples that appear realistic in overall multivariate structure but deviate from real data at the level of individual genes. This discrepancy is consistent in both datasets and might contribute to reduced performance in downstream evaluations that depend on gene-wise distributions such as co-expression and differential expression recovery, which are examined in the subsequent sections.

Deep generative models (Embedded Diffusion, CVAE variants, WGAN-GP) attain the highest coverage while showing density values well above 1 across both datasets. This may suggest that synthetic samples broadly span the real manifold and fall within densely populated real regions. DP models, in contrast, exhibit near-zero density and coverage especially in the TCGA-COMBINED dataset, implying that samples lie largely outside the real data manifold.

Classifier-based discriminative performance (F1) and relative distance-to-closest mostly follow similar ordering across models. Deep generative DP-models, including DP-CTGAN, and DP-CVAE, tend to generate samples farther from any individual real record while simultaneously being more easily separable from real data by a classifier (indicated by high discriminative score, implying low indistinguishability). MVN achieves the lowest discriminative score across both datasets, illustrating that its synthetic samples are particularly difficult to distinguish from real data. MVN also lies in the high-fidelity region of both MMD and KL, which suggests strong global distributional alignment despite its simple parametric form. Among deep generative models without formal privacy guarantee, the Embedded Diffusion model attains the highest fidelity levels.

### Downstream predictive utility of synthetic bulk RNA-seq datasets

Utility refers to the extent to which a synthetic dataset can replicate the performance of real data in a downstream task, as well as how well it preserves the critical features underlying predictive decisions. We assessed this using the “train-on-synthetic, test-on-real” (TSTR) scheme, where the models trained on synthetic data were evaluated on the held-out real test data compared to the models trained on real data tested on the held-out real data (TRTR).

In this study, the downstream tasks are molecular subtype prediction for the TCGA-BRCA dataset and cancer type prediction for the TCGA-COMBINED dataset. Performance is summarized using AUC-ROC and F1 reported as relative performance (TSTR/TRTR), with ratios closer to one indicating higher utility. We additionally report the important feature overlap proportion between real and synthetic trained models, using the top predictive genes from each class, aggregated globally (Methods).

Table 2 shows that AUC-ROC and F1 relative scores exhibit highly consistent patterns across datasets. MVN achieves the best performance in all utility metrics across both datasets. CVAE, CVAE-GMM and Embedded Diffusion achieve substantially higher relative scores across most metrics in both datasets. These same models also exhibit the highest important feature overlap proportions, indicating better preservation of the genes that are relevant for the downstream classification task. In contrast, CTGAN variants, together with P-NMF and DP-CVAE display the lowest relative scores across most metrics, which points to substantial loss of downstream predictive signal when trained on synthetic data. We also note that DP-PGM consistently outperforms other formally DP models and achieves relative scores that are comparable to, or slightly lower than, those of non-DP models. However, its proportion of overlapping important features is substantially lower than that of non-DP models across both datasets.

**Table 2:** Utility metrics across TCGA-BRCA and TCGA-COMBINED datasets. We report the mean of each metric across five folds, with standard deviation in parentheses. Directionality arrows (↓ ↑) indicate the direction of better performance. Relative (rel.) metrics report the performance of the synthetic data relative to real data. For each metric, the best-performing model (and any model within one standard deviation of it) is shown in bold, and the next-best is underlined. Bolding and underlining are determined within each dataset independently; the two datasets are shown together for compactness and are not directly compared

	MVN	CVAE	DP-CVAE ε = 4	CTGAN	DP-CTGAN ε = 50	EMB-Diffusion	NMF	P-NMF	DP-PGM ε = 4	WGAN-GP	CVAE-GMM
**TCGA-BRCA**											
**F1 (rel.) ↑**	**1.001 (0.015)**	0.982 (0.039)	0.474 (0.065)	0.328 (0.071)	0.310 (0.083)	0.949 (0.029)	0.830 (0.053)	0.289 (0.098)	0.852 (0.023)	0.938 (0.022)	0.976 (0.012)
**AUC-ROC (rel.) ↑**	**0.988 (0.003)**	**0.986 (0.007)**	0.655 (0.021)	0.517 (0.015)	0.389 (0.008)	0.985 (0.006)	0.872 (0.049)	0.532 (0.060)	0.943 (0.012)	0.968 (0.010)	0.983 (0.005)
**Feature overlap ↑**	**0.341 (0.049)**	0.272 (0.076)	0.033 (0.011)	0.056 (0.017)	0.014 (0.022)	**0.302 (0.054)**	0.072 (0.023)	0.064 (0.038)	0.040 (0.020)	0.137 (0.033)	**0.318 (0.042)**
**TCGA-COMBINED**											
**F1 (rel.) ↑**	**0.999 (0.003)**	0.994 (0.003)	0.777 (0.021)	0.086 (0.025)	0.083 (0.034)	0.993 (0.003)	0.854 (0.056)	0.366 (0.057)	0.908 (0.024)	0.983 (0.004)	0.992 (0.005)
**AUC-ROC (rel.)↑**	**0.999 (0.001)**	0.998 (0.001)	0.893 (0.008)	0.514 (0.022)	0.523 (0.064)	**0.999 (0.001)**	0.970 (0.015)	0.749 (0.015)	0.993 (0.002)	0.997 (0.001)	0.997 (0.001)
**Feature overlap ↑**	**0.471 (0.037)**	0.405 (0.046)	0.129 (0.029)	0.147 (0.029)	0.120 (0.023)	0.370 (0.043)	0.165 (0.046)	0.165 (0.046)	0.175 (0.038)	0.241 (0.026)	0.368 (0.030)

### Differential expression, co-expression and pathway recovery across synthetic RNA-seq datasets

We investigated how well the synthetic data captures gene expression dependencies via differential expression, co-expression and pathway recovery patterns.

#### Differential expression (DE)

**Fig. 2:**
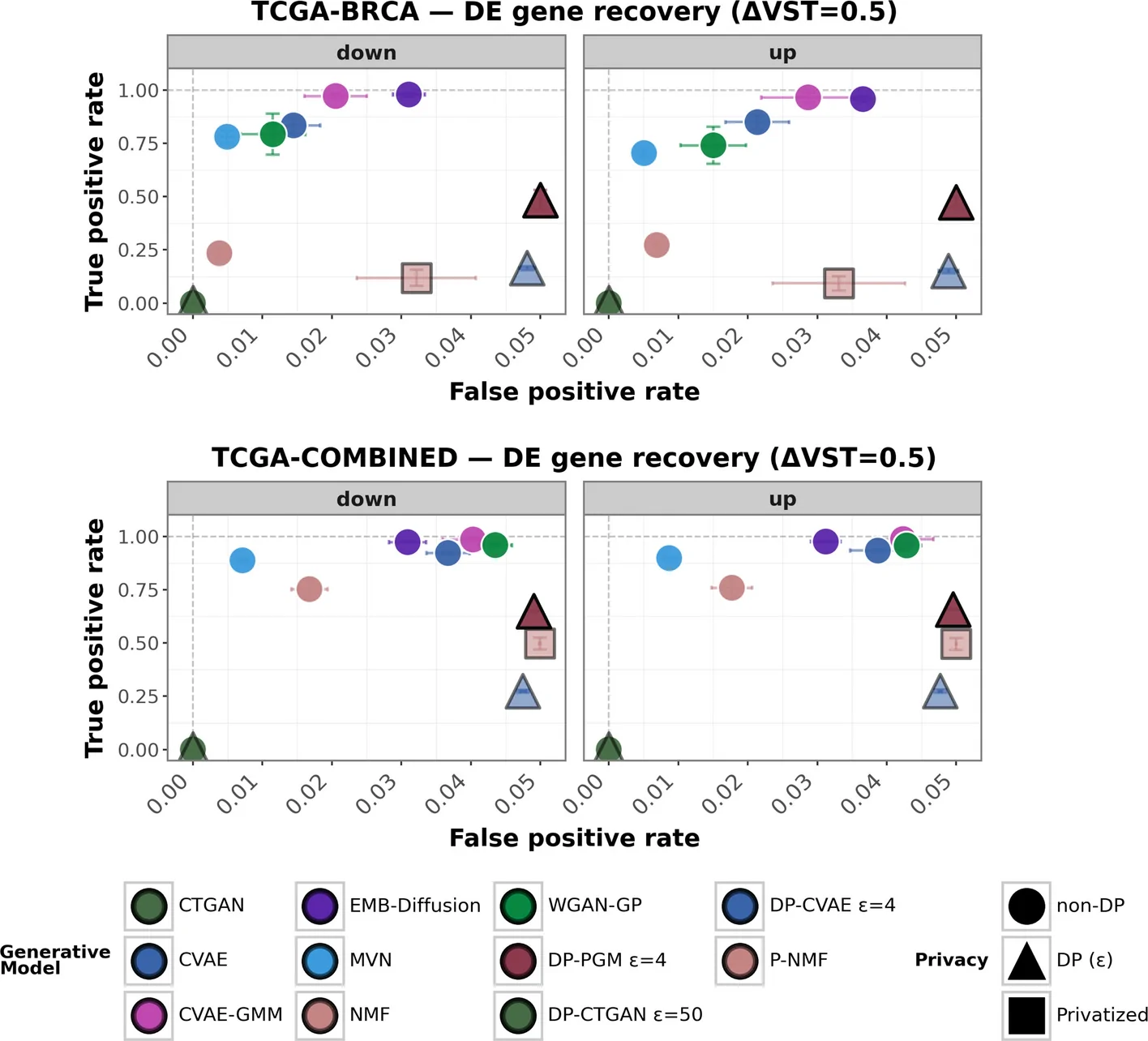
Differential Expression (DE) recovery analysis for TCGA-BRCA and TCGA-COMBINED datasets. It is reported at an effect-size threshold of ΔVST = 0.5 (genes with effect size exceeding 0.5 are called differentially expressed), shown as true positive rate (TPR) against realized false positive rate (FPR). The FPR axis is restricted to ≤ 0.05 to focus on the high-specificity regime; generators exceeding this are clamped to the axis edge and flagged. Each coloured point is the mean across five folds for one generative model, with error bars showing the standard deviation along each axis. Versions of the same model share a base colour and are distinguished by marker shape indicating privacy category: circles for non-DP, triangles for formally DP, and a square for the partially private P-NMF method. Models appear toward the top-left of the ROC-style axes when recovery is strong (high TPR, low FPR)

We evaluated DE recovery using paired comparisons between groups (e.g., molecular subtypes for TCGA-BRCA and cancer types for TGCA-COMBINED; see Methods). Figure 2 shows DE recovery under stringent effect-size criterion (ΔVST > 0.5), averaged across comparisons and folds, illustrated as true positive rate (TPR) against false positive rate (FPR) in a ROC-style format, with the FPR axis restricted to ≤ 0.05. Up- and down-regulated genes are assessed separately to capture directional differences. TPR performance per pairwise comparison in the TCGA-BRCA and TCGA-COMBINED datasets is shown in Additional file 3: Figs. S4-S5.

Across both datasets, up- and down-regulation patterns are broadly similar, with model rankings largely consistent between directions. Deep generative models CVAE-GMM and Embedded Diffusion achieve the highest DE recovery, followed closely by CVAE and WGAN-GP. Among statistical models, MVN attains the highest TPR and generally lower FPR than deep models. DP-PGM, while exhibiting higher TPR than other DP-constrained models in both datasets, remains less successful than non-DP models with lower TPR and generally higher FPR. CTGAN variants collapse to the origin (TPR = FPR = 0) across both datasets, recovering no differential expression, consistent with their poor utility performance.

In ablation experiments across effect-size thresholds (ΔVST = 0.0, 0.2, 0.5, 1.0; Additional file 3: Fig. S6), a consistent trend emerges: most models move toward lower realized FPR as the effect-size threshold increases. This trend holds within each dataset; at stringent thresholds (≥ 0.5), however, realized FPR levels are higher in the larger TCGA-COMBINED dataset than in TCGA-BRCA, particularly for deep generative models. This may possibly reflect greater size and biological heterogeneity of the TCGA-COMBINED dataset. NMF variants, which show poor DE TPR in BRCA, improve substantially in the TCGA-COMBINED dataset.

Overall, consistent with their high downstream utility, expressive deep generative models achieve strong DE recovery under stringent effect-size control, while simpler parametric models such as MVN also perform well. DP constraints generally reduce DE recovery TPR, particularly for subtle DE signals, as reflected in lower TPR levels on TCGA-BRCA (molecular subtypes) compared to TCGA-COMBINED (cancer types).

#### Gene co-expression

**Fig. 3:**
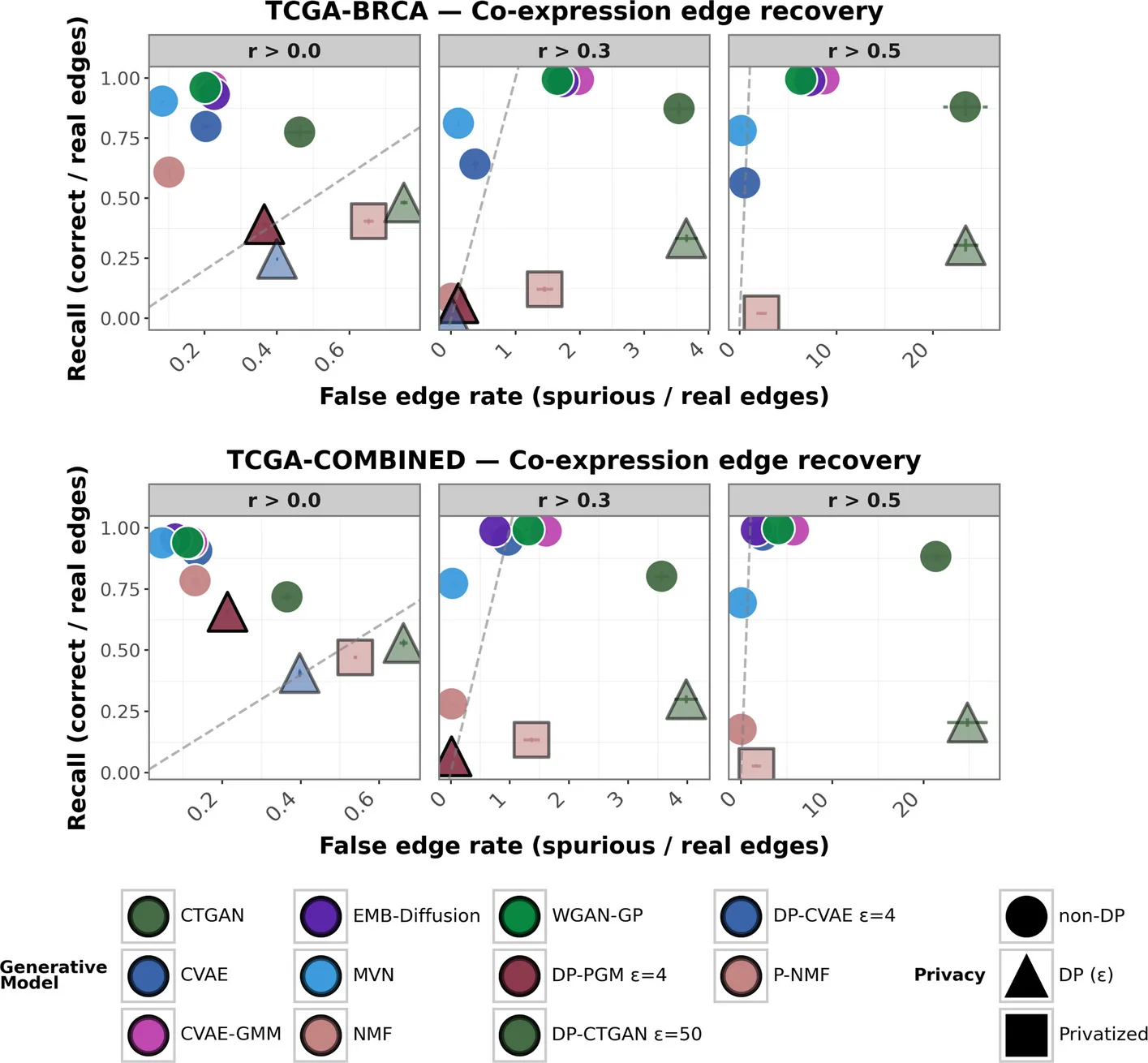
Co-expression recovery analysis for the TCGA-BRCA and TCGA-COMBINED datasets. Each facet corresponds to the correlation threshold applied to gene–gene pairs. Each colored point represents the mean metric values for a generative model across five folds, with error bars indicating the corresponding standard deviation along each axis. Versions of the same model share a base colour and are distinguished by marker shape indicating privacy category: circles for non-DP, triangles for formally DP, and a square for the partially private P-NMF method.The y-axis shows the true positive rate (fraction of real edges recovered in the synthetic network), and the x-axis shows the false edge rate (spurious edges in the synthetic network, normalized by the number of true edges in the real network). The gray dashed line indicates a reference linear trend

We assessed co-expression recovery by comparing gene–gene correlation networks between real and synthetic data under correlation thresholds implemented in hCoCena package [39] (Methods). In order to assess recovery across the range of biologically meaningful correlation strengths, we evaluate cutoffs *r* > 0, *r* > 0.3, and *r* > 0.5 which correspond to permissive (any correlation), moderate, and strong correlations respectively under standard conventions. Higher true positive rates indicate stronger preservation of biological co-expression patterns, whereas false edge rates reflect spurious relationships introduced by the generative model. False edge rate is computed as the number of spurious edges in the synthetic network, normalized by the number of true edges in the real network (Fig. 3).

In the TCGA-BRCA dataset, increasing the correlation threshold (0.0 to 0.3 to 0.5) challenges several models. At *r* > 0.5, DP-PGM, DP-CVAE, and NMF produce no co-expression edges at all (i.e. a flat network) indicating their synthetic data contains no strongly correlated gene pairs. This reflects a loss of strong pairwise dependency structure where these models retain weaker co-expression (edges at *r* > 0.3) but cannot reproduce the strong relationships present in real data. This can be explained differently for each model: for DP-PGM, because it reconstructs only the low-dimensional marginals released under differential privacy; for DP-CVAE, because gradient noise decorrelates features; and for NMF, because low-rank reconstruction averages out fine correlation structure. On the other hand, CTGAN variants persist at *r* > 0.5. Rather than losing strong correlations, it generates gene pairs with high but spurious correlation absent from the real network. While the false-edge rate is normalized by the (shrinking) number of real edges at higher thresholds, inflating all methods' rates, CTGAN variants’ rates far exceed the remaining methods at both *r* > 0.3 and *r* > 0.5. This indicates genuine fabrication of spurious high-correlation edges that survive the strong (*r* > 0.5) correlation threshold.

Thus, at stringent thresholds models fail in different ways: flat networks that lose real dependency structure (DP-PGM, DP-CVAE, NMF) and dense networks that generate false dependencies (CTGAN variants). Deep generative models (CVAE-GMM, Embedded Diffusion, WGAN-GP) maintain relatively high TPR at the stricter cutoff but introduce substantially more spurious edges. MVN preserves a very low false-edge rate across cutoffs, though its TPR drops as the threshold tightens.

A broadly similar pattern is also observed in the TCGA-COMBINED dataset. As the correlation cutoff increases, DP-CVAE and DP-PGM again fail to reliably recover the real co-expression network at r > 0.3 and r > 0.5, respectively. CVAE-GMM, Embedded Diffusion and WGAN-GP consistently exhibit high TPR while still introducing a high number of spurious edges, even though the two datasets have comparable numbers of real edges at this threshold. This might reflect the increased stability of correlation estimates in greater sample size and more faithful generation at larger sample size.

These results illustrate a trade-off: deep generative models can produce dense networks that preserve many true edges but also generate many spurious interactions, whereas simpler models like MVN favor sparse networks with fewer false edges but may lose some true connections under stringent thresholds. This balance is further influenced by dataset characteristics.

#### Pathway analysis

Pathway recovery was evaluated using single-sample GSEA (ssGSEA) [40] of the 50 Molecular Signatures Database (MsigDB) [41] Hallmark gene sets (Methods). Table 3 summarizes pathway preservation across the evaluated generative models and datasets using four complementary metrics: the mean Kolmogorov–Smirnov (KS) statistic, which measures differences between the distributions of pathway scores in real and synthetic data; the mean absolute standardized effect which quantifies shifts in pathway activity, the geometric mean ratio assessing agreement in pathway variability; and the fraction of pathways with FDR-significant KS tests (Frac. sig.), representing the fraction of pathways with enrichment score distributions significantly different between real and synthetic data.

**Table 3:** Pathway recovery performance across TCGA-BRCA and TCGA-COMBINED datasets. The results summarize the ssGSEA pathway enrichment score comparisons for the 50 MSigDB Hallmark gene sets. Best (bold) and runner-up (underline) models are determined within each dataset separately. Directionality arrows (↓ ↑) indicate the direction of better performance. (KS: Kolmogorov–Smirnov; Frac. sig.: fraction of significant KS tests)

	MVN	CVAE	DP-CVAE ε = 4	CTGAN	DP-CTGAN ε = 50	EMB-Diffusion	NMF	P-NMF	DP-PGM ε = 4	WGAN-GP	CVAE-GMM
**TCGA-BRCA**											
**Mean KS ↓**	**0.075 (0.012)**	0.154 (0.023)	0.314 (0.012)	0.280 (0.014)	0.720 (0.027)	0.116 (0.014)	0.118 (0.018)	0.445 (0.014)	0.183 (0.017)	0.161 (0.025)	0.179 (0.028)
**Mean |effect| ↓**	**0.090 (0.044)**	0.291 (0.064)	0.681 (0.032)	0.601 (0.054)	1.413 (0.107)	0.203 (0.025)	0.179 (0.064)	1.072 (0.063)	0.327 (0.058)	0.293 (0.073)	0.343 (0.069)
**Var ratio → 1**	1.254 (0.057)	0.787 (0.031)	0.884 (0.062)	0.630 (0.084)	0.028 (0.011)	1.099 (0.093)	0.797 (0.085)	1.599 (0.089)	0.681 (0.030)	0.826 (0.043)	0.733 (0.045)
**Frac sig (FDR) ↓**	**0.444 (0.135)**	0.868 (0.023)	0.996 (0.009)	0.984 (0.009)	1.000 (0.000)	0.748 (0.050)	0.804 (0.050)	1.000 (0.000)	0.948 (0.033)	0.900 (0.028)	0.892 (0.036)
**TCGA-COMBINED**											
**Mean KS ↓**	**0.051 (0.002)**	0.086 (0.006)	0.232 (0.015)	0.214 (0.013)	0.731 (0.047)	**0.050 (0.004)**	0.056 (0.003)	0.225 (0.010)	0.132 (0.006)	0.060 (0.006)	0.078 (0.008)
**Mean |effect| ↓**	0.082 (0.006)	0.132 (0.013)	0.489 (0.048)	0.376 (0.027)	1.689 (0.427)	0.080 (0.013)	**0.021 (0.008)**	0.466 (0.020)	0.229 (0.020)	0.075 (0.013)	0.129 (0.017)
**Var ratio → 1**	1.122 (0.008)	0.788 (0.015)	0.709 (0.033)	0.981 (0.017)	0.028 (0.010)	1.008 (0.039)	0.758 (0.013)	1.007 (0.040)	0.742 (0.017)	0.955 (0.036)	0.860 (0.033)
**Frac. sig (FDR) ↓**	0.856 (0.026)	0.992 (0.011)	1.000 (0.000)	1.000 (0.000)	1.000 (0.000)	**0.628 (0.067)**	0.788 (0.091)	1.000 (0.000)	0.988 (0.011)	0.880 (0.058)	0.928 (0.054)

Across both datasets, MVN and Embedded Diffusion show similar profiles with small absolute mean effect size (0.1–0.2) and variance ratio modestly above 1 (1.1–1.25), indicating faithful pathway-score means with mildly inflated spread. For MVN, this inflation may partly reflect the additive Gaussian noise (σ = 0.5) applied during generation rather than the model's fit alone. NMF also performs consistently across both datasets, with a small effect size and variance ratio below 1, indicating faithful pathway-score centers with mild under-dispersion.

The effect-size and variance ratio together also distinguish different failure modes. DP-CTGAN has a near-zero variance ratio (0.028 in both datasets) with a large effect size (> 1.4), indicating collapse to a near-constant distribution. DP-CVAE shows a moderate effect size (0.4–0.7) with variance ratio below 1 (0.7–0.9), demonstrating under-dispersion together with a location shift. P-NMF shows variance ratio at or above 1 (1.0–1.6) with moderate-to-large effect size (0.4–1.0) which suggests preserved-to-inflated spread with a location shift. This contrasts with NMF's faithful, mildly under-dispersed profile, isolating privatized training as the source of the distortion. This location shift is also visible in the signed per-pathway effect-size heatmaps (Additional file 3: Figs. S7-S8): most generators share a positive directional effect for a group of gene sets, whereas P-NMF and DP-CVAE show the opposite (negative).

Fraction of pathways with significant distributional shift (frac. sig.) is higher in the TCGA-COMBINED dataset than TCGA-BRCA; however, this metric is sensitive to the number of samples. The larger TCGA-COMBINED dataset provides greater power in each pathway's KS test to detect real-synthetic distributional differences, leading more of the (fixed 50) pathways to reach significance. The increase in frac. sig. therefore largely reflects sample size rather than worse fidelity.

### Privacy risk under membership inference attacks (MIA)

We evaluated the privacy vulnerability of the synthetic datasets under a black-box adversarial setting without query access to the generator. The adversary is assumed to have access to the released synthetic dataset and a set of candidate records comprising both members (training samples used to fit the generator) and non-members (held-out real test samples), but no access to the generator's parameters, gradients, or internal representations. Attacks score each candidate's membership using only the released synthetic data (and, for reference-anchored attacks, an auxiliary reference set). Membership inference risk was assessed separately for each fold under five-fold cross-validation (Methods).

We adopted multiple black-box membership inference attacks that require access only to the released synthetic data (see Methods): distance- (Monte Carlo (MC) [42], GAN-leaks, GAN-leaks calibrated [37]), density-estimation- (DOMIAS [43]), and classifier-based (LOGAN-D1 [44], Confidence LR, Confidence RF) attacks (see Additional file 2: Table S2 for details). Some of these attacks are reference-anchored which means that they use an auxiliary held-out real reference set that mimics the training distribution (DOMIAS, LOGAN, GAN-leaks calibrated). These were evaluated only on TGCA-COMBINED, as TCGA-BRCA's small size precluded holding out a reference set. Because DOMIAS's density estimation is infeasible at the original dimensionality (978 landmark genes), all reference-anchored attacks were evaluated under four dimensionality-reduction approaches for comparability: principal component analysis (PCA), supervised differentially expressed genes (SDEG), discriminative genes (DG), and differentially variable genes (DVG) (Methods, Additional file 2: Table S3 for details).

We emphasize that the empirical privacy risks reported here hold specifically under this threat model; low measured risk indicates resistance to the attacks evaluated in this setting and does not imply broader privacy guarantees against stronger adversaries (e.g. model-access or white-box attacks). We also note that aggregate metrics such as AUC-ROC reflect average-case vulnerability across individuals. Prior work [45, 46] shows that the most vulnerable individuals can be substantially more exposed than the average, therefore low average vulnerability does not guarantee protection for every individual. The MIA performances are reported with a threshold-independent metric (AUC-ROC) and an operational metric (TPR at FPR = 0.1). Complete MIA results are provided in Additional file 3: Figs. S9-S14.

**Fig. 4:**
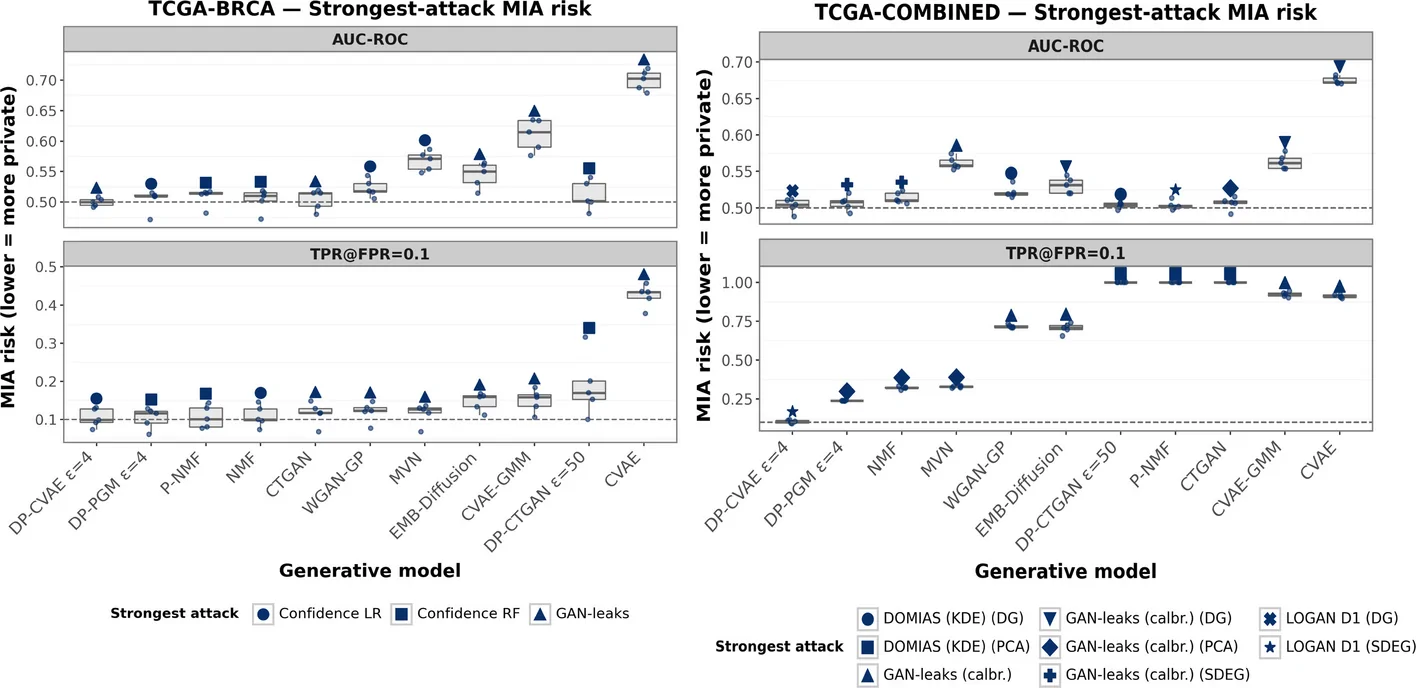
Membership inference attack (MIA) performance on the TCGA-BRCA and TCGA-COMBINED datasets. We report two complementary privacy-risk metrics in separate panels: area under the ROC curve (AUC-ROC) and true positive rate at a fixed false positive rate of 0.1 (TPR@FPR = 0.1); for both, higher values indicate greater privacy risk (random-guessing baselines: AUC-ROC = 0.5, TPR@FPR = 0.1). For each generative model, we report the. the performance of the most successful attack, with the marker shape indicating which attack that is. Each boxplot summarizes that attack's performance across the five cross-validation folds

Figure 4 demonstrates, for each generative model, membership inference risk under the best performing attack (i.e. the attack achieving the highest MIA risk). Across attacks and datasets, three distinct privacy regimes emerge: random guessing level (i.e. near the AUC-ROC = 0.5 and/or TPR@FPR = 0.1 baseline), moderate and elevated vulnerability.

#### Random-guessing level attack performance

Formally differentially private (DP) methods, including DP-CVAE $\left(\in =4\right)$, and DP-PGM $\left(\in =4\right)$ generally operate close to random-guessing level (TPR@FPR = 0.1 ≈ 0.1 and AUC-ROC ≈ 0.5). This pattern is particularly stable for DP-CVAE across datasets and metrics. DP-PGM, on the other hand, exhibits elevated TPR@FPR = 0.1 under the calibrated GAN-leaks attack (PCA) in the TCGA-COMBINED dataset while still exhibiting lower MIA risk than the rest of the non-DP generative models. This highlights the importance of DP guarantees in adversarial settings that include auxiliary reference data. It’s also important to note that, although these models satisfy the same formal DP guarantee and provide the same theoretical upper bound on the attack performance, DP is applied at different stages in these models, for instance, gradients (DP-CVAE) and probabilistic parameters (DP-PGM). Therefore, this difference might influence how privacy constraints interact with specific attack algorithms.

Among non-DP methods, NMF also exhibits lower vulnerability across settings. Given its constrained factorization structure and comparatively moderate utility, this suggests that limited model expressiveness may inherently restrict the amount of membership-relevant signal encoded in the synthetic data. A cautionary case is CTGAN variants, which exhibit near random-guessing level membership inference risk in AUC-ROC metric across both datasets. These methods simultaneously demonstrate poor downstream utility, DE and co-expression recovery. Therefore, their apparent resistance to MIA may primarily reflect limited learning of the underlying data structure rather than privacy protection.

#### Moderate attack vulnerability

MVN, Embedded Diffusion, and WGAN-GP occupy an intermediate vulnerability regime by AUC-ROC (up to 0.57). Under the calibrated GAN-leaks, which utilizes an auxiliary dataset, WGAN-GP and Embedded Diffusion exhibit substantially higher TPR@FPR values than MVN (TPR@FPR = 0.1 up to 0.72), whereas MVN demonstrates higher AUC-ROC of the three. Despite its parametric simplicity, MVN’s explicit modeling of global co-variance structure may encode sufficient distributional information to enable membership inference above random-guessing level. We can suggest that moderate vulnerability is not restricted to deep neural networks, but may also arise from faithful modeling of high-dimensional structure.

#### Elevated attack vulnerability

CVAE and CVAE-GMM exhibit systematically elevated vulnerability across both datasets, with CVAE showing the highest risk across most attack-metric combinations (TPR@FPR = 0.1 up to 0.92, AUC-ROC up to 0.91). Under GAN-leaks, which emphasizes proximity between synthetic and real samples, reconstruction- or posterior-sampling-based models are particularly susceptible. This effect is further amplified under the calibrated GAN-leaks attack in the TCGA-COMBINED dataset, where auxiliary reference data increases attack power. Interestingly, CVAE-GMM, despite re-using real data to fit Gaussian mixtures, is not consistently more vulnerable than CVAE. This might indicate that data re-use might not immediately result in a higher MIA risk, as the GMM may regularize generation by sampling from cluster-level latent distributions rather than directly reflecting individual posterior encodings.

A notable pattern also emerges with DOMIAS under PCA reduction, which yields near-perfect (1.0) attack performance on CTGAN, DP-CTGAN, and P-NMF under TPR@FPR = 0.1. These are precisely the models that collapse to degenerate synthetic distributions (low fidelity and utility performances). DOMIAS's density-ratio attack trivially separates members when the synthetic distribution is severely mis-specified, so this saturation flags generator collapse rather than a conventional privacy leak.

#### Comparison of membership inference attack methods

Across attacks and datasets, no single membership-inference attack is uniformly strongest; the most effective attack depends on the dataset, the generator, and the privacy metric. In the TCGA-BRCA dataset, the attack achieving the highest risk at the random-to-moderate level varies, comprising a mix of classifier- and distance-based attacks, while at the elevated level GAN-leaks consistently ranks highest across both privacy metrics. In the TCGA-COMBINED dataset, highest-risk attack is dominated by reference-anchored methods (LOGAN, DOMIAS, and GAN-leaks calibrated), indicating that auxiliary reference data meaningfully increases membership-inference power. The two privacy metrics favor different feature-selection variants: under TPR@FPR, GAN-leaks calibrated with the full feature set (no selection) produces the highest leakage, whereas under AUC-ROC, GAN-leaks calibrated with discriminative-gene selection (DG; top-100 genes separating synthetic from reference) is strongest. The impact of feature selection on individual reference-anchored attacks, and the agreement of feature-selection variants across metrics, are provided in Additional file 3: Figs. S15-S17. Under AUC-ROC, feature selection does not reorder the vulnerability ranking of generators; under TPR@FPR, however, PCA disagrees with the full feature set and SDEG (supervised DE genes, 100-genes) approaches. LOGAN is unaffected by any feature-selection method, as it performs near random-guessing level and thus carries no vulnerability signal for feature selection to reorder.

We also assess whether the raw nearest-neighbor distance (distance-to-closest) could serve as a simpler privacy proxy in place of attack-based risk (Additional file 3: Fig. S18). Its association with membership-inference leakage varies in sign and strength across attacks and metrics. It is generally negatively correlated with AUC-ROC which is the expected direction for a valid proxy (greater distance, lower leakage); however, under TPR@FPR the sign varies across attacks and datasets. The association is strongest for distance-based attacks, as both rely on nearest-neighbor distance, but is inconsistent for other attack types. Overall, distance-to-closest is not a reliable standalone privacy proxy since its relationship to leakage depends on the attack type and metric. Recent work similarly notes that proximity alone may not reliably indicate meaningful privacy risk [47].

### Trade-offs and complementarity between evaluation metrics

**Fig. 5:**
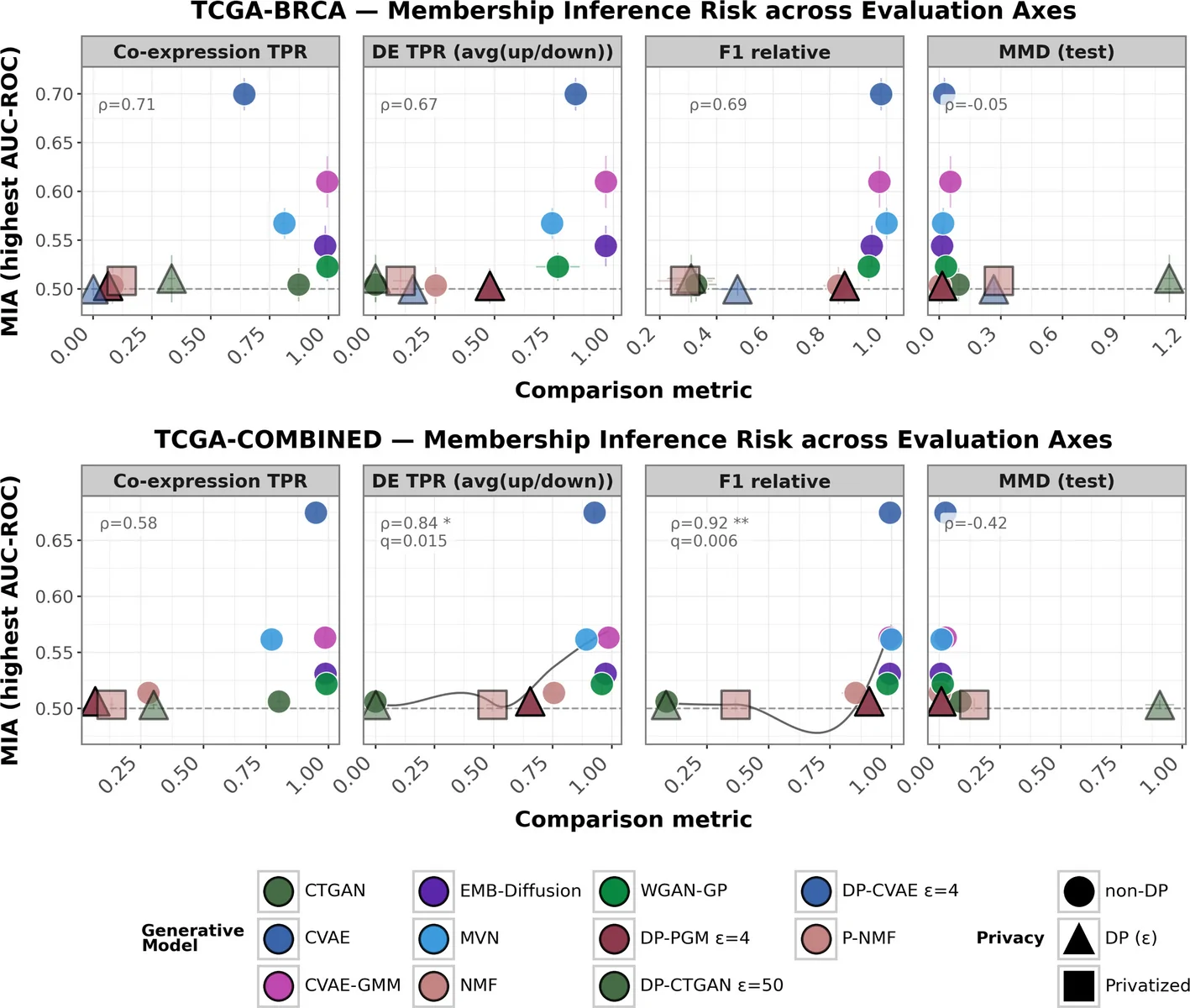
Membership inference risk across evaluation axes.Best-performing membership inference attack (MIA) risk is quantified as AUC-ROC where for each generator, the highest AUC-ROC is selected across all evaluated attacks (higher indicates greater privacy risk). Each panel shows the relationship between MIA risk and a representative metric from another evaluation axis: relative F1 (utility), MMD (fidelity), DE recovery TPR and co-expression recovery TPR (biological plausibility). Higher values indicate better quality for F1 and the recovery metrics; lower MMD indicates better fidelity. When two metrics show a statistically significant Spearman correlation (Benjamini/Hochberg FDR-adjusted q < 0.05), a non-parametric LOESS trend line is overlaid, and the corresponding q-value is annotated. Versions of the same model share a base colour and are distinguished by marker shape indicating privacy category: circles for non-DP, triangles for formally DP, and a square for the partially private P-NMF method. (*: *q* < 0.05, **: *q* < 0.01; DE threshold: ΔVST=0.5; co-expression threshold *r *> 0.3)

**Fig. 6:**
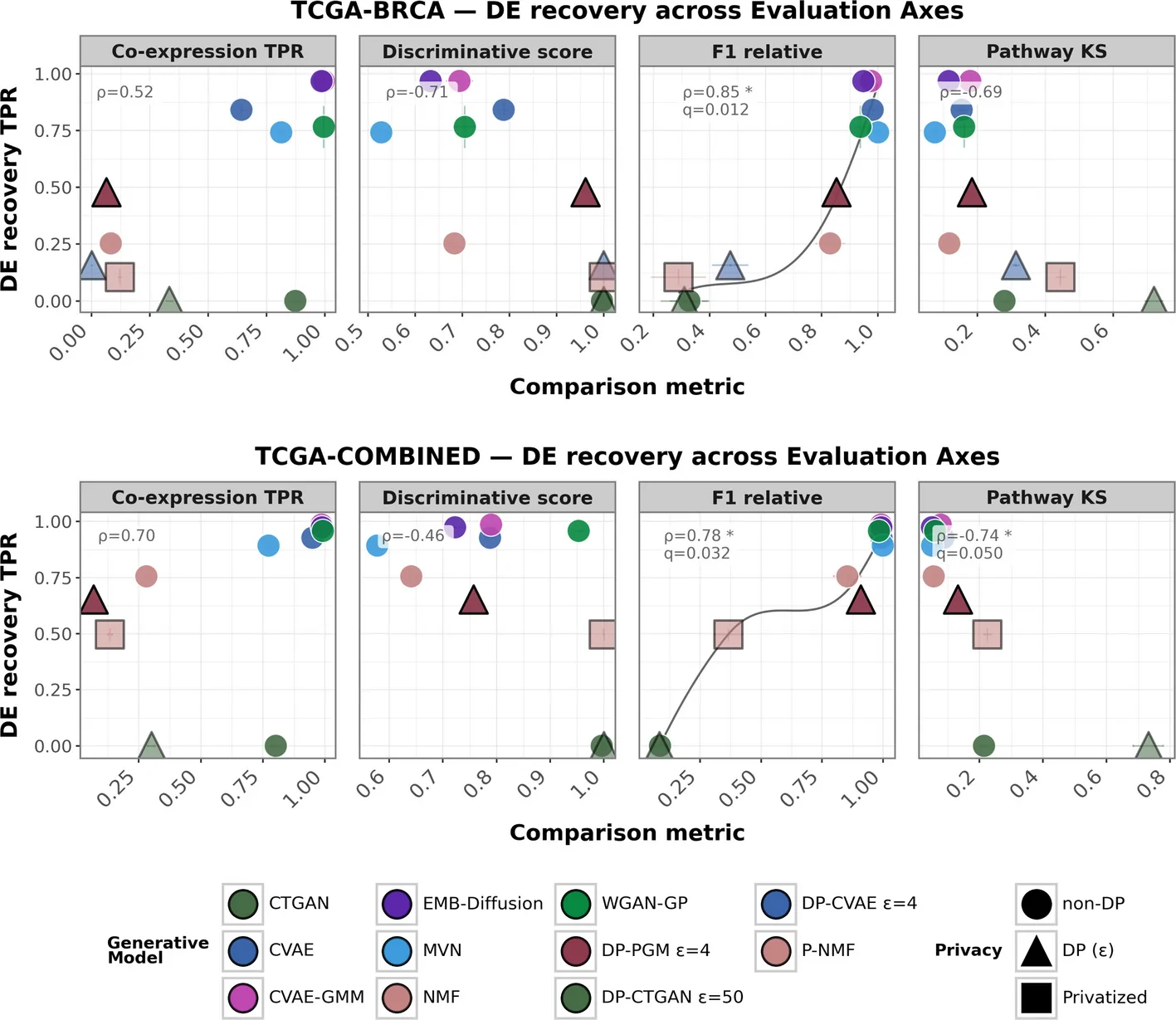
DE recovery across evaluation axes. DE recovery is the average true positive rate (TPR) of up- and down-regulated gene recovery (ΔVST > 0.5). Each panel shows the relationship between DE recovery and a representative metric from another axis: relative F1 (utility), discriminative score (fidelity), and co-expression recovery TPR and pathway KS (biological plausibility). Directions differ by metric: higher is better for DE recovery, F1, and co-expression TPR; lower is better for discriminative score and pathway KS. When two metrics show a significant Spearman correlation (Benjamini/Hochberg FDR-adjusted q < 0.05), a non-parametric LOESS trend line is overlaid, and the q-value is annotated. Versions of the same model share a base colour and are distinguished by marker shape indicating privacy category: circles for non-DP, triangles for formally DP, and a square for the partially private P-NMF method. (*: *q *< 0.05, **: *q* < 0.01; DE threshold: ΔVST=0.5; co-expression threshold *r* > 0.3)

**Fig. 7:**
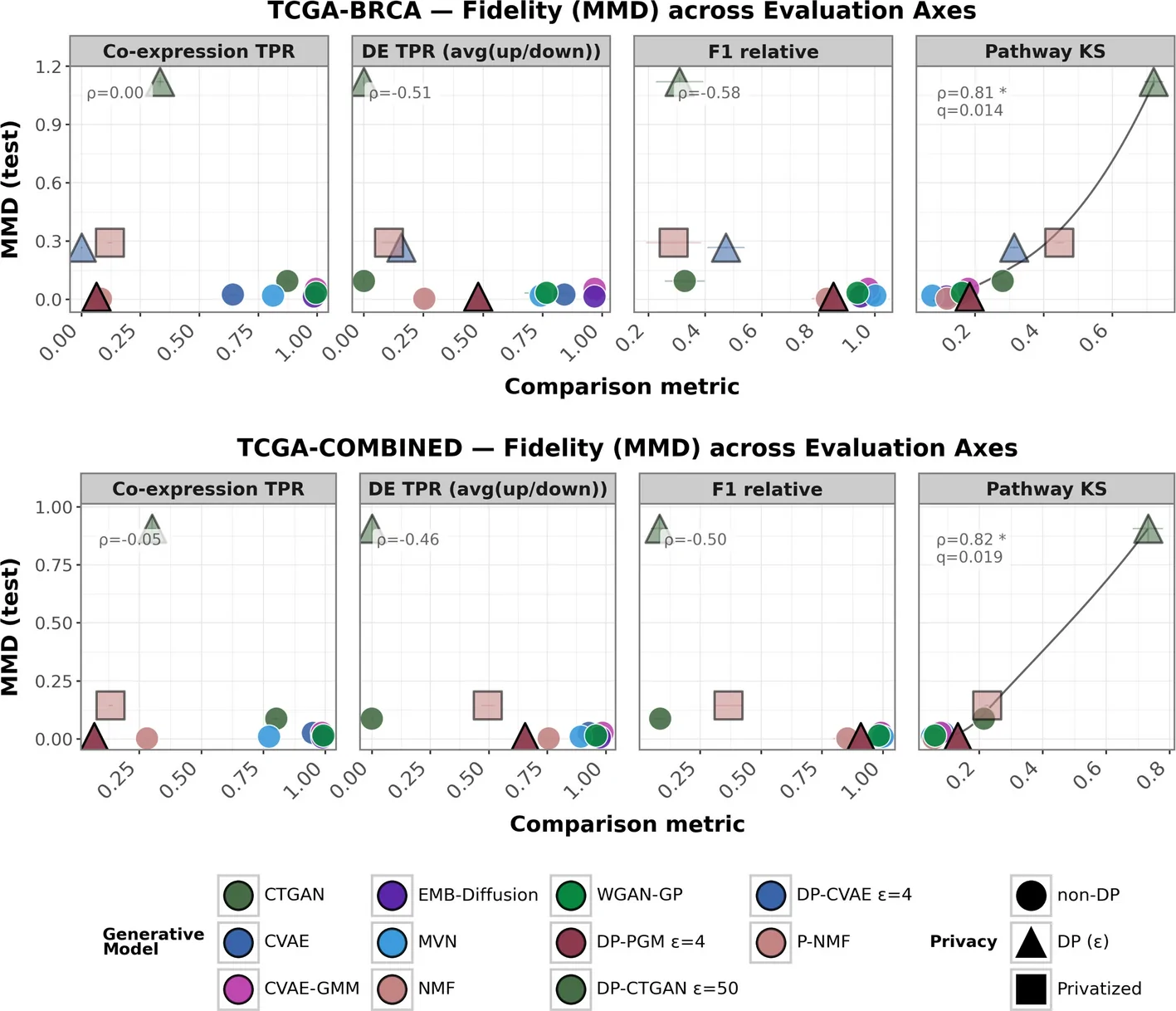
MMD (test) across evaluation axes. Each panel shows the relationship between MMD (test), a representative distributional-fidelity metric, and a representative metric from another evaluation axis (relative F1, DE recovery TPR, co-expression recovery TPR, pathway KS). Lower MMD indicates better distributional fidelity; metric directions differ by axis (↑ better for F1 and co-expression/DE TPR, ↓ better for pathway KS). When two metrics show a statistically significant Spearman correlation (Benjamini/Hochberg FDR-adjusted q < 0.05), a non-parametric LOESS trend line is overlaid, and the corresponding q-value is annotated. Versions of the same model share a base colour and are distinguished by marker shape indicating privacy category: circles for non-DP, triangles for formally DP, and a square for the partially private P-NMF method. (*: *q *< 0.05, **: *q* < 0.01; DE threshold: ΔVST=0.5; co-expression threshold *r* > 0.3)

Here we investigate trade-offs between evaluation axes and how they interact. We focus on three key relationships: (i) membership inference risk against other evaluation axes, examining whether privacy-preserving methods sacrifice performance (Fig. 5); (ii) differential expression recovery against other axes, assessing whether biological plausibility aligns with other quality metrics (Fig. 6); and (iii) distributional fidelity against downstream utility, examining whether statistical similarity translates to downstream predictive performance (Fig. 7).

We analyze correlations separately for each dataset given differences in attack calibration and data properties (*n* = 11 methods, Spearman correlation). Correlations presented in the following trade-off analyses are extracted from a comprehensive correlation matrix across all (raw) evaluation metrics (11 selected metrics, 55 pairwise comparisons; full matrix in Additional file 3: Figs. S19-S20), with global FDR correction $\left(\alpha =0.05\right)$ applied across all comparisons. When two metrics show a statistically significant Spearman correlation (FDR-adjusted q < 0.05), a non-parametric LOESS trend line (span = 1.0, no confidence band) is overlaid, and the corresponding q-value is annotated.

#### Membership inference risk across evaluation axes

We first examine whether privacy risk trades off against the other evaluation axes. Figure 5 summarizes the relationship between membership inference risk under each generator’s best performing attack (MIA AUC-ROC), and representative metrics from other evaluation axes: relative F1 (utility), MMD (fidelity), DE recovery TPR (ΔVST > 0.5), biological plausibility) and co-expression recovery TPR (*r* > 0.3, biological plausibility).

Since DE recovery is highly consistent across both up- and downregulated genes (Fig. 2), subsequent analyses report DE recovery based on average TPR across both directions. Across utility and biological plausibility metrics higher scores indicate better synthetic data quality; higher MIA AUC-ROC corresponds to increased privacy risk and lower MMD means higher fidelity. For the trade-off analysis, we utilize AUC-ROC metric for MIA since it provides a graded, saturation-robust measure better suited to quantifying the continuous relationship between privacy risk and other evaluation axes. The operational TPR@FPR view is reported in the membership-inference risk subsection (Fig. 4).

Across both datasets, we observe positive associations between membership inference risk (AUC-ROC) and both F1 relative utility and DE recovery (significant in TCGA-COMBINED dataset with q<0.05), and with co-expression recovery also exhibiting a substantial but non-significant positive correlation $\left(\rho>0.5\right)$. At the model level: deep-learning-based methods with high utility and DE recovery, including CVAE variants, WGAN-GP, and Embedded Diffusion, as well as MVN, exhibit elevated MIA risk (AUC-ROC > 0.5).

The DP-model DP-PGM remains close to random guessing performance (AUC-ROC ~ 0.5), while achieving moderate to high relative utility and DE preservation but very limited co-expression recovery. Notably, s P-NMF and DP-PGM exhibit higher DE recovery and relative utility in the larger TCGA-COMBINED dataset (TPR > 0.5) than TCGA-BRCA, while maintaining random-guessing level attack performance (~ 0.5) in both datasets. This observation is consistent with the prior literature suggesting that the utility loss caused by DP mechanisms decreases when the size of the data increases [48, 49]. Notably, co-expression recovery for these models do not improve with dataset size, suggesting that while individual gene-level signals benefit from more data, network-level relationships remain difficult to capture under DP-constraints. The same privacy–utility asymmetry holds as the privacy budget is varied directly: sweeping ∈ (1, 4, 10, 50) within DP families generally increases utility and DE recovery, monotonically in the larger COMBINED dataset, while empirical MIA risk stays near random-guessing, rising only marginally with ∈ (Additional file 3: Fig. S1). This reflects the gap between DP's worst-case theoretical guarantee, which weakens with ∈, and realized empirical leakage.

Overall, these results indicate that the privacy–utility trade-off is not universally observed. While deep-learning-based non-DP methods that achieve high downstream utility and biological plausibility frequently exhibit elevated MIA risk, certain approaches, such MVN and DP-PGM, demonstrate that competitive utility and biological preservation can be achieved with moderate to low privacy risk. This non-universality is why privacy must be assessed directly rather than inferred from utility or fidelity. We therefore recommend reporting risk under the best-performing attack per model (i.e. aggregating over attacks by taking the maximum) for privacy auditing, as no quality metric reliably predicts empirical leakage.

#### Differential expression (DE) recovery across evaluation axes

We next examine how DE recovery relates to the other axes, to clarify whether it can serve as a proxy for them. In Fig. 6, we correlate DE TPR with representative metrics: co-expression recovery TPR and pathway KS core (biological plausibility), relative F1 (utility), and MMD (fidelity). For co-expression recovery TPR and F1 utility, higher values indicate better synthetic data quality, while for MMD and pathway KS lower values indicate better quality. DE recovery (average) is quantified as the average true positive rate (TPR) of up- and down-regulated genes at an effect size threshold of ΔVST = 0.5, with higher values indicating better preservation of biologically meaningful expression differences.

Across both datasets, DE recovery is strongly positively associated with downstream utility $\left(q<0.05\right)$, indicating that synthetic data preserving key DE genes retain the predictive signals required for the downstream task. Both downstream tasks (i.e. molecular subtype and cancer type prediction) are primarily driven by between-class mean expression differences. Consequently, synthetic datasets that more faithfully recover DE genes retain the dominant discriminative signals required for classification, resulting in higher relative F1. In the TCGA-BRCA dataset only, DE recovery exhibits high negative correlation with discriminative score, indicating better DE recovery accompanies lower distinguishability between synthetic and real data; however, this association does not reach significance in the larger TCGA-COMBINED dataset.

DE recovery also exhibits a moderate-to-high but non-significant positive correlation with co-expression TPR across both datasets and strong negative correlation (q ~ 0.05) with pathway KS score in TCGA-COMBINED dataset. The co-expression association, though positive in direction, does not reach significance at the available number of generators.

These findings indicate that, for the evaluated tasks, DE preservation is predictive of downstream utility and tracks pathway-level fidelity in the larger dataset but not co-expression (network) structure. This suggests that gene-level, network-level, and functional-level biological signals are partially distinct and no single biological metric substitutes for the others. We therefore recommend evaluating biological plausibility at each level separately: DE for gene-level signal, co-expression for network structure, and pathway analysis for functional coordination.

#### Fidelity across evaluation axes

Finally, in Fig. 7, we examine how MMD (test), a representative distributional-fidelity metric, relates to the other evaluation axes; namely: representative metrics from utility (relative F1) and biological plausibility (co-expression and DE recovery TPR, pathway KS score). We note that MMD captures the distributional family of fidelity; the manifold-based metrics (density, coverage) behave differently, aligning more with utility (Additional file 3: Figs. S19-S20).

MMD (test) shows a strong positive correlation with pathway KS across both datasets $\left(q<0.05\right)$; as both are oriented as lower-is-better which suggests better global distributional alignment accompanies better pathway-level recovery. Correlations with DE TPR and relative F1 are negative but non-significant, directionally consistent with distributional fidelity tracking biological and downstream quality without reliably predicting it. Therefore, within the context of evaluated models, global distributional fidelity relates most closely to pathway-level fidelity among the other evaluation axes examined. Because global distributional fidelity does not on its own guarantee gene- or task-level quality, and manifold-based fidelity metrics (density, coverage) relate to the evaluation axes differently (Additional file 3: Figs. S19-S20; discussed further in Discussion), we recommend including both distributional and manifold-based metrics in quality evaluation rather than relying on a single fidelity metric.

## Discussion

This study provides a systematic, multi-dimensional evaluation of synthetic bulk RNA-seq generators, emphasizing the tension and complementarity between distributional realism, downstream utility, biological plausibility and privacy.

### Evaluation metrics are complementary, not interchangeable

Across two cancer datasets with distinct sizes and biological heterogeneity, and spanning diverse generative model families with total 11 approaches, our results indicate that no single evaluation metric provides a sufficient assessment of synthetic RNA-seq data quality. Instead, fidelity, utility and biological plausibility capture complementary, and often non-overlapping properties of generative model performance.

Utility metrics are strongly and consistently correlated with each other, reflecting a coherent notion of predictive-signal preservation (Additional file 3: Figs. S2-S3). Manifold-based fidelity metrics (density and coverage) cluster with the utility metrics, showing moderate positive correlations (except density (train)). MMD, KL divergence, and discriminative score form a separate cluster and show moderate-to-high negative correlations with utility, showing that better distributional similarity accompanies higher utility. The two fidelity families are themselves only weakly correlated, indicating they track different aspects of the data. Although fidelity and utility are therefore partially aligned, they remain distinct axes: no fidelity metric fully substitutes for direct utility evaluation, and the two fidelity families capture different properties from one another. Fidelity is thus not monolithic, and fidelity and utility are not interchangeable.

Biological plausibility metrics separate into distinct dimensions. DE recovery (both up- and down-regulated) correlates significantly with downstream utility (positively) and pathway KS (negatively), suggesting that preserving strong transcriptional signals improves predictive performance and pathway fidelity. However, this association is task-dependent: because the evaluated downstream tasks primarily rely on marginal gene expression shifts, DE recovery is an effective proxy for predictive signal retention. For tasks driven more by gene regulation or network topology, co-expression preservation may be more important. Co-expression recovery TPR shows moderate but non-significant correlations with utility and DE recovery, indicating only tentative links to gene-level signals, but correlates significantly with manifold-based fidelity metrics. This suggests that co-expression primarily reflects preservation of higher-order dependency structure.

These findings demonstrate that evaluation outcomes are jointly shaped by generative model architecture, dataset characteristics, metric selection, and the downstream task for which the synthetic data are intended. Performance is therefore not an intrinsic property of a model alone, but emerges from the interaction between model assumptions and task-specific signal structure. Accordingly, benchmarking synthetic RNA-seq generators requires a multi-dimensional framework that evaluates biological preservation across complementary levels of biological organization (from gene- and pathway-level signals to higher-order co-expression structure) alongside distributional fidelity and downstream utility. Limiting assessment to fidelity or predictive performance alone risks overlooking biologically meaningful distortions that may not immediately manifest in classifier-based metrics.

### Membership inference risk is model- and attack-dependent

The privacy evaluation reveals a clear tradeoff between model expressiveness and membership risk. Models that closely approximate the underlying data distribution, particularly deep expressive generative models, tend to exhibit elevated vulnerability, whereas formally DP-constrained methods help suppress detectable membership signals. At the same time, low empirical vulnerability can also arise from limited structural learning, as underfitted generative models can exhibit reduced membership risk simply because they fail to capture meaningful data signals. Therefore, privacy risk must be interpreted in conjunction with utility and fidelity metrics to avoid mistaking underfitting as robustness.

Differences across attack algorithms, especially when auxiliary reference data are introduced, further demonstrate that measuring vulnerability depends not only on the generative model but also on the adversarial setting.

Finally, the results also show that non-DP models are not uniformly risky. Simpler statistical approaches can occupy intermediate or low-risk regimes, suggesting that they can serve as meaningful privacy-utility baselines when benchmarking more expressive architectures.

### Architecture-level patterns and failure modes across generative models

Across model families, performance differences are primarily driven by architectural inductive biases and regularization strategies, and how each model interacts with high-dimensional continuous data rather than model class alone. Variational models (CVAE and CVAE-GMM) showed strong utility and recovery of biologically meaningful signals. In particular, CVAE-GMM outperformed the standard CVAE in the smaller TCGA-BRCA dataset, by coupling representation learning with density modeling via a mixture prior. The Embedded Diffusion model achieved performance comparable to CVAE-GMM across utility, biological and privacy metrics, suggesting that diffusion architectures may offer a favorable balance between expressivity and regularization.

Adversarial models showed more variable outcomes: WGAN-GP achieved a strong performance in several evaluation axes, whereas CTGAN and DP-CTGAN often underperformed, reflecting architectural assumptions tailored to mixed tabular data (e.g. feature-wise transformations and PAC-based discrimination) that might interact poorly with high dimensional continuous VST-transformed RNA-seq data. DP-CTGAN further incurred performance penalties due to privacy constraints, likely the cost of per-feature preprocessing in high dimensional settings.

Among formal DP methods, DP-PGM achieved the strongest overall DP-compliant performance, however with reduced utility and biological plausibility relative to non-DP deep neural methods. This may be because the model explicitly encodes only 1-way and 2-way marginals; correlations between genes not included in these marginals are under-represented in the synthetic data, reducing biological plausibility for analyses that rely on gene–gene interactions. MVN, a simpler structure method, performed consistently well across evaluation axes, and maintained moderate privacy risk, illustrating that less expressive but well-regularized architectures can deliver robust performance.

Overall, these results show that privacy, fidelity, utility and biological plausibility are governed by architectural inductive biases: highly expressive models with regularization favor fidelity at the cost of privacy, whereas structured or regularized models trade coverage for robustness.

### Dataset heterogeneity and size changes trade-off patterns

This benchmark evaluates two datasets with distinct structural and statistical properties: the smaller TCGA-BRCA dataset (~ 1 K samples) comprising multiple molecular subtypes, and the larger, multi-tissue TCGA-COMBINED dataset (~ 5 K samples) spanning diverse cancer tissues. The downstream task is inherently more challenging in TCGA-BRCA, where molecular subtypes within a single cancer could be subtly distinct, whereas multi-class cancer type prediction in TGCA-COMBINED generally yields higher baseline performance.

Across evaluation dimensions, dataset size and heterogeneity modulate the observed trade-offs. For example, deep generative models show largely consistent privacy risk rankings across both datasets. TCGA-COMBINED additionally enabled reference-anchored attacks (via held-out reference dataset), providing a more realistic adversarial assessment that was not possible for the smaller TCGA-BRCA dataset.

Biological metrics further reflect dataset-dependent effects. For Differential Expression (DE) recovery, most non-CTGAN models achieve higher and more stable results in TCGA-COMBINED. Pathway analysis is also influenced by dataset size. The fraction of pathways with a significant distributional shift (frac. sig.) is higher in TCGA-COMBINED than TCGA-BRCA, but this increase largely reflects greater statistical power in each pathway's KS test at the larger sample size rather than worse fidelity, and is therefore not directly comparable across datasets.

In summary, these findings suggest that dataset scale, heterogeneity and task structure influence the magnitude and stability of observed fidelity, utility, biological preservation, and privacy trade-offs, and emphasize that conclusions drawn from benchmarking studies might not transfer directly across datasets with differing biological and statistical complexity.

### Practical implications for users

Our benchmarking suggests that both metric choice and model choice should be guided by the intended aim, as no single metric or model performs best across all axes.

For evaluation, we recommend matching metrics to the property of interest: highest-performing membership-inference attack (per generative model) for privacy auditing; downstream-task metrics (F1, AUC-ROC, feature overlap) for task-relevant deployment; distributional distances (MMD, KL) together with manifold-based similarity (density, coverage) for distributional and manifold fidelity; and the DE, co-expression, and pathway metrics for gene-level, network-level, and functional biological structure respectively.

For model selection: for downstream analyses requiring accurate differential expression or co-expression recovery, CVAE-GMM and Embedded Diffusion provide strong performance (with WGAN-GP a further option if well-tuned), though deep models can introduce spurious co-expression edges at stringent thresholds. Where privacy is a priority, DP-PGM achieves low membership-inference risk under most attacks, but at the cost of reduced fidelity and co-expression recovery. MVN offers surprisingly strong utility with fast training and can serve as a useful baseline before training more complex deep generative models. No single model performs best across all axes; MVN (a simple baseline), Embedded Diffusion (a strong deep model), and DP-PGM (a privacy-preserving approach) provide useful reference points.

Overall, downstream task, preprocessing, dataset size, and heterogeneity strongly affect outcomes, so users should weigh these factors jointly when selecting both metrics and a generative model.

## Challenges and limitations

The scope of this benchmark is limited to bulk RNA-seq data derived from cancer-relevant (TCGA) gene expression datasets with comparatively modest sample sizes (~ 1,000 and ~ 5,000), and a curated set of 978 landmark genes. Therefore, the observed performance of the generative models reflect their behavior within this reduced feature space and should not be directly extrapolated to complete transcriptome setting, where higher dimensionality, sparsity, and complex gene–gene interactions may pose additional challenges. Moreover, all datasets were preprocessed using variance-stabilizing normalization (VST), which limits the generalizability of the results to models trained on raw count data or alternative normalization schemes.

The dataset size constrains the range of applicable models, as some architectures require larger cohorts for stable optimization or are unsuitable in high-dimensional settings. The relatively small number of evaluated methods (*n* = 11) further restricts the comparative scope, particularly when examining cross-metric correlations under limited statistical power. Accordingly, the findings should be viewed as cohort-specific and method-dependent, inclusion of additional approaches may refine and modify the comparative conclusions.

Although we incorporate a broad range of fidelity, utility, biological and privacy metrics, the evaluation is necessarily incomplete. Biological preservation is assessed through biological pathway, differential expression and co-expression recovery under fixed conditions, including effect size threshold and correlation thresholds. Alternative thresholds or biological analyses may result in different relative rankings of the generative models. Therefore, all conclusions should be interpreted as conditional on the selected metrics and evaluation settings rather than as universal statements about model performance.

Privacy evaluation presented here encompasses seven membership inference attacks spanning distance-based, density-based, and classifier-based methodological categories evaluated under a black-box, offline threat model. This includes DOMIAS-KDE, a density-ratio estimator representing one of the strongest attack classes applicable under our threat models, evaluated across multiple feature selection categories. Stronger threat models such as gray-box or white-box settings in which the adversary exploits knowledge of the model architecture, or attempts to infer model parameters from the synthetic outputs, as well as shadow-model-based likelihood-ratio tests (e.g. LiRA [50]), fall outside the black-box, output-only setting we define. Such settings can largely be avoided in practice by withholding the generative model, and extending the analysis to them remains an avenue for future work.

Finally, our membership-inference setup assumes the adversary's candidate profiles share the training data's representation, without the measurement noise, partial coverage, or platform differences that arise in practice. While standard transcriptomic normalizations (e.g. VST, log, TPM) are widely shared, an informed adversary would plausibly apply a matching transformation. However, these transformations are fit to dataset-specific parameters and would not reproduce an identical representation from independently measured auxiliary data. Therefore, our shared-representation assumption is favorable to the adversary. We further do not model cross-run, cross-platform, or cross-assay measurement mismatch. Our evaluation is therefore best understood as a model for continuous, relatively homogeneous biological data processed under a common pipeline. Extending membership inference to noisy, partial, or cross-platform auxiliary measurements would give a more realistic risk assessment and is an important direction for future work.

### Community engagement lessons

This benchmarking study builds on the CAMDA 2025 Health Privacy Challenge, yielding several lessons relevant to future community benchmarks in synthetic transcriptomics. First, leaderboard-style ranking is not fitted to multi-objective evaluation. No method performed consistently well across fidelity, utility, biological preservation and privacy risk, or across datasets. Encouraging accompanying extended abstracts helped surface both strengths and failure modes beyond single-score comparisons. Future benchmark efforts might consider providing example visualizations, such as heatmaps, or trade-off plots, to help participants interpret multi-metric performance and understand trade-offs better.

Second, evaluating only a single, optimized model snapshot can mask important sensitivities. Participant feedback showed that privacy and utility often vary with architectural and hyperparameter choices, suggesting that apparent model performance may reflect tuning rather than intrinsic behavior. Benchmark platforms should encourage transparent reporting of hyperparameters, privacy budgets, and architectural variants, and allow multiple submissions to enable robust comparison across model configurations.

Finally, fragmented tooling hindered biological evaluation in participants’ extended abstracts. Analyses such as co-expression and differential expression recovery were underutilized, likely because R-based tools required specific libraries or dependencies. Future benchmarks should ensure smooth cross-language integration, for example via containerized pipelines, so participants can run all metrics without skipping analyses due to language or dependency barriers.

Overall, these observations highlight the importance of emphasizing trade-offs over rankings, encouraging exploration of model variants rather than single submissions, and providing tightly integrated, cross-domain evaluation pipelines in future benchmarks.

### Future direction

Building on our joint effort with DECIDER [51] and the DREAM challenges [52], we are currently assessing the generalizability of the methods evaluated in this benchmark by generating synthetic ovarian cancer data and validating its quality on the ovarian cancer patients in the DECIDER cohort. This replication is intended to be part of the OpenEBench platform (https://openebench.bsc.es/), explicitly improved to support benchmarking. Following steps should be testing the models across even larger and more heterogeneous datasets, including single-cell RNA-seq, which was also one of the tracks of the CAMDA 2025 Health Privacy Challenge, as well as multi-omics and non-cancer electronic health records. In parallel, development of generative models for data synthesis could benefit from multi-objective optimization, explicitly balancing utility, biological plausibility and privacy rather than treating these as post-hoc evaluations.

Synthetic data utility and privacy may vary across demographic or clinical subpopulations, but our analyses are reported only at the dataset-level. Prior work has shown disparate impacts of differential privacy [47] and membership inference attacks [53]. Future benchmarking should assess synthetic data generation under sensitive attribute stratification (e.g. race, gender) to enable fairness-aware evaluation and more realistic assessment.

Transformer-based and large language model (LLM) approaches are increasingly explored for synthetic biomedical and omics data generation [14, 54, 55] illustrating the growing use of pretrained, high-capacity models that integrate biological or clinical priors. Evaluating such models is a natural extension of this work. The multi-axis framework used here which jointly assesses distributional fidelity, downstream utility, biological plausibility, and privacy applies directly to them. Their scale and reliance on pretraining may, however, introduce distinct, model-specific failure modes across all of these axes. For instance, pretraining priors could improve biological plausibility while introducing subtle distributional artifacts, and memorization of pretraining data could create privacy-leakage pathways not captured by generator-only threat models. Systematic, multi-axis evaluation will therefore be increasingly important as these models are adopted.

Finally, privacy evaluation could be expanded. Beyond the aggregate membership-inference risk reported in this study, per-record (per-individual) membership vulnerability warrants dedicated analysis, as the most exposed individuals can be substantially more vulnerable than the average. Privacy assessment could also extend beyond membership inference to include re-identification, linkage, attribute inference, or model inversion attacks [32, 56–58]. Differential privacy mechanisms should be systematically studied to disentangle the effects of privacy accounting, noise injection strategies and model architecture on empirical trade-offs. Collectively, these directions can enhance the robustness, fairness, and interoperability of synthetic data benchmarking in transcriptomics.

## Conclusions

In this work, we benchmarked generative models for private and biologically meaningful synthetic data generation, building on the community competition, CAMDA 2025 Health Privacy Challenge. Our results demonstrate that, within transcriptomics context, the quality of synthetic data must be evaluated across multiple complementary axes, as no single metric can capture biological signal recovery and utility. These findings highlight the importance of multi-dimensional benchmarking frameworks for synthetic data generation, as evaluation outcomes are jointly shaped by generative model architecture, dataset characteristics, metric selection, and the downstream task for which the synthetic data are intended. Consequently, this evaluation framework serves as a guidance for transcriptomics and broader healthcare applications and emphasizes that task-specific benchmarking should become a standard practice before data sharing.

## Methods

### Challenge aim and design

The Health Privacy Challenge CAMDA 2025 (https://benchmarks.elsa-ai.eu/?ch=4 [34]) aimed to benchmark generative models for private and biologically plausible synthetic transcriptomics data generation. The challenge was established with two tracks: Track 1 focused on bulk RNA-seq data and Track 2 focused on single-cell RNA-seq data (OneK1K [59]). Track 1 ran in two complementary tasks that are referred to as Blue Team versus Red Team, sharing the bulk RNA-seq dataset but diverging in goals.

- The Blue Teams generated synthetic bulk RNA-seq datasets that balance the utility and privacy trade-off.
- The Red Teams designed methods to launch a membership inference attack (MIA) on synthetic datasets to determine whether the target sample is included in the training dataset that generated the synthetic dataset.

In this benchmarking study, we only cover the baseline generative methods (provided by the organizers) and the valid participant-submitted generative models (Track 1, Blue Team). For the membership-inference privacy assessment, we use only the attack methods provided by the organizers and do not include participant-submitted attacks (Track 1, Red Team), as no valid submissions were received. All reported privacy results derive solely from the black-box attack methods described in the Evaluation Framework subsection.

### Datasets and pre-processing

We re-distributed the open access The Cancer Genome Atlas (TCGA) [60] bulk gene-expression data (STAR counts) obtained from the Genomic Data Commons (GDC) portal (https://portal.gdc.cancer.gov) using the TCGABiolinks R package [61]. We provided two preprocessed datasets, that we refer to as TCGA-BRCA and TCGA-COMBINED.

- TCGA-BRCA: The dataset contains 1094 (donor) samples with known subtypes from breast cancer (TCGA-BRCA) cohort, belonging to either LumA, LumB, Basal, Her2, or Normal.
- TCGA-COMBINED: The dataset consists of 5222 primary tumor (donor) samples containing 12 cancer types spanning 10 tissue types: Breast (TCGA-BRCA), Colorectal (TCGA-COAD), Esophagus (TCGA-ESCA), Kidney (TCGA-KIRC, TCGA-KIRP), Liver (TCGA-LIHC), Lung (TCGA-LUSC, TCGA-LUAD), Ovarian (TCGA-OV), Pancreatic (TCGA-PAAD), Prostate (TCGA-PRAD), and Skin (TCGA-SKCM).

The raw count data for both datasets is preprocessed similar to [22]:

- Each donor contributed a single sample.
- For TCGA-BRCA, the samples without known molecular (sub)types are filtered.
- Low count genes are filtered: genes with counts ≥1 in at least 10% of samples are kept.
- Remaining genes are normalized using the DeSeq2 variance-stabilizing transformation (VST) transformation with blind = FALSE [62].
- Analysis was restricted to the LINCS L1000 landmark gene set comprising 978 genes [63] that are reported to capture most transcriptional variance and enable the inference of the remaining genes in transcriptome (https://clue.io/command?q=/gene-space%20lm, accessed on Nov 1, 2024).

TCGA COMBINED dataset was subsequently divided into two stratified sets, comprising 4398 and 824 donors/samples. The smaller set, 824 records, was reserved for launching membership inference attack algorithms that require an external dataset. Consequently, only the larger dataset was employed to generate and evaluate synthetic data.

All generative models were trained using VST-transformed real expression data and generated synthetic datasets directly in the VST-transformed expression space, restricted to the 978 LINCS L1000 landmark genes.

### Experiment design

TCGA bulk RNA-seq datasets, denoted as D, were used to generate synthetic datasets using a generative model f(D). Each dataset was split into a stratified five-fold cross validation setting using a fixed random seed [42] to enable comparisons across models. For each fold $i\in \{\mathrm{1,2},\mathrm{3,4},5\}$, a shape-matched synthetic dataset ${{D}_{SYN}}^{(i)}$ was generated from the model f(D) trained on the corresponding training partition ${{D}_{TR}}^{(i)}$. The quality of the synthetic data was evaluated on the corresponding held-out test set ${{D}_{TE}}^{(i) }$ and performance metrics were averaged across the five folds to obtain a robust estimate of generalization. This cross-validation procedure also produced five membership inference datasets, one per fold, in which members correspond to training samples and non-members correspond to test samples.

### Differential Privacy (DP)

DP is a mathematical definition of privacy, which roughly indicates that including or removing a sample from the dataset does not significantly affect the outcome of an analysis [1]. A method M is (ϵ,δ)-differentially private if, given two datasets of ${D}_{1}$ and ${D}_{2}$ that differ by at most one element, it holds: $P[M({D}_{1})\in S] \le {e}^{\epsilon }P[M({D}_{2})\in S] + \delta$ where S is a subset of outputs generated by M, ϵ is a non-negative parameter that controls the “privacy budget” and δ is a non-negative parameter that indicates the level of privacy guarantee being violated. Determining ϵ and δ involves a trade-off between utility and privacy. For instance, smaller ∈ indicates stronger privacy, allowing more noise, and therefore might lead to less accurate results in utility. DP can be layered onto several paradigms (factorization, PGMs, VAEs/GANs), typically by perturbing gradients, statistics, or outputs. The benefit is formal privacy guarantees; the cost is reduced fidelity as ∈ shrinks.

In the datasets used in this study, each patient contributes a single expression profile (see Datasets and Preprocessing), therefore, a record corresponds to a patient. Record-level (add/remove) adjacency therefore protects the presence or absence of an individual patient, and we use "record-level" and "patient-level" interchangeably below.

### Generative models

Here we describe the methods included in this benchmarking study in model family groups. Although different taxonomical approaches for generative models are used [64], here we group models according to their underlying generative mechanisms (statistical baselines, latent-variable autoencoders, adversarial models, etc.) and focus on the subset of approaches that were instantiated and evaluated in this benchmark.

Baselines were curated by the benchmark organizers to provide reference performance for each model family and were publicly available during the challenge. These include Multivariate Normal Distribution (MVN), Conditional Variational Autoencoder (CVAE) [65, 66], and Conditional Tabular Generative Adversarial Network (CTGAN) [67], where CVAE and CTGAN were implemented with and without Differential Privacy (DP) [1]. In addition, two post-challenge methods, CVAE with Gaussian Mixture Model (CVAE-GMM) [68] and Wasserstein GAN with Gradient Penalty (WGAN-GP) [69, 70] were included to increase the diversity of evaluated methods.

Submissions were provided by external participants and reflected realistic generative modeling practices. These include a Diffusion Model with noise injection [71], Differentially Private-PGM (DP-PGM) [72], and implementation of Non-negative Matrix Factorization (NMF) [73] with and without privatization step (P-NMF).

#### 1. Statistical and sampling based

These models specify a parametric probability distribution and sample from it, assuming relatively simple dependence structures and provide interpretable baselines, highlight the performance of classical probabilistic assumptions, and serve as a reference point for more complex generative models. Here we detail the instances evaluated in this benchmark.

##### 1.1 Multivariate Normal (MVN) model

The Multivariate Normal (MVN) model assumes a joint Gaussian distribution, where the mean vector and covariance matrix capture pairwise correlations among genes. In this benchmark, we adopted a class-conditional MVN, fitting separate models for each subtype or disease type to serve as a simple and interpretable statistical baseline.

**Implementation details:** For each subtype (/disease type) *s*, the empirical mean vector ${\widehat{\mu }}_{s}$ and covariance matrix ${\widehat{\Sigma }}_{s}$ were computed from training data. To ensure numerical stability, the covariance was symmetrized and projected to a positive semi-definite matrix by clipping negative eigenvalues and adding a small diagonal jitter ∈I. Synthetic samples were then drawn as ${x}_{syn}\sim N\left({\widehat{\mu }}_{s}, {\widehat{\Sigma }}_{s}^{\mathrm{P}\mathrm{S}\mathrm{D}}\right)$ and perturbed with additive multivariate Gaussian noise ${x}_{\mathrm{s}\mathrm{y}\mathrm{n}}^{\mathrm{n}\mathrm{o}\mathrm{i}\mathrm{s}\mathrm{y}}={x}_{syn}+\eta ,\eta \sim N\left(0,\alpha {\widehat{\Sigma }}_{s}^{\mathrm{P}\mathrm{S}\mathrm{D}}\right)$ where α=0.5 controls the noise scale. This additive noise simulates potential real-world measurement variability while preserving the underlying correlation structure of the data. We investigated noise levels of 0.5, 1, and 5 to systematically explore the effect of increased variance on downstream analyses, and reported the resulting impact on differential expression recovery in Additional file 3: Fig. S21.

Limitations: The MVN assumption restricts the model to Gaussian features and linear correlations, which may not capture multimodality, over-dispersion, or nonlinear dependencies in gene expression. 

##### 1.2 Non-negative matrix factorization (NMF)

The NMF model represents data as low-rank latent factors [73] and is widely used in bioinformatics to decompose datasets into interpretable structures, such as metagenes and sample clusters to provide biological insights [74]. This model was submitted by a participant of the challenge.

**Implementation:** Normalized counts were filtered to obtain non-negative integer matrices. The input expression matrix $V\epsilon {\mathbb{R}}_{\ge 0}^{p\times g}$ (patients × genes) is factorized into non-negative matrices $W\in {\mathbb{R}}_{\ge 0}^{p\times k}$ and $H\in {\mathbb{R}}_{\ge 0}^{k\times g}$ using MiniBatchNMF from Scikit-learn, which employs a coordinate descent solver and computes updates in batches for scalability: V≈WH.

The NMF rank k=50 was determined via Frobenius-norm reconstruction error elbow criterion. Latent encodings in *W* capture metagene activity and are clustered via K-means to form biologically coherent subgroups. The number of clusters *k*_*c*_ was dynamically set equal to the number of classes in input data. For each cluster *c*, per gene statistics were computed, mean (${\mu}_{c}$), variance (${\sigma}_{c}^{2}$), and zero-fraction (*z*_*c*_), to parameterize a Gaussian sampler that reconstructs synthetic count profiles while preserving realistic dispersion and sparsity patterns.

To preserve class identity, a Random Forest classifier (${n}_{\mathrm{t}\mathrm{r}\mathrm{e}\mathrm{e}}=100$) was trained on the real latent encodings *W*, using Gini impurity to measure the quality of a split. Synthetic labels were assigned probabilistically by sampling from the posterior class probabilities predicted by this classifier.

**Limitations:** The linear additive structure of NMF may miss complex, nonlinear dependencies between genes or samples.

##### 1.3 Privatized-non-negative matrix factorization (P-NMF)

P-NMF follows the same modeling framework as NMF approach but adds calibrated Gaussian noise to the per-cluster gene means. The synthetic data are then drawn from these privatized summaries, but do not carry a formal differential-privacy (DP) guarantee.

**Implementation:** The model first decomposes the non-negative matrix $V\in {\mathbb{R}}_{\ge 0}^{p\times g}$ (patients x genes) into latent factors, V≈WH where $W\in {\mathbb{R}}_{\ge 0}^{p\times k}$ and $H\in {\mathbb{R}}_{\ge 0}^{k\times g}$ are estimated using MiniBatchNMF. Privacy protection is achieved through a single Gaussian mechanism applied to the released per-cluster gene-means. The NMF factorization and clustering are non-private internal steps and consume no budget. Each sample's contribution is bounded by a per-dataset constant *R* (*R* about 369 for TCGA-BRCA, *R* about 361 for TCGA-COMBINED), which sets the sensitivity of a cluster mean to *R* divided by the cluster size. Neighboring datasets differ by the removal of one patient's expression profile (set to zero). Gaussian noise scaled to this sensitivity (with ∈ and a fixed $\delta ={10}^{-5}$) is added to the per-cluster gene means. All other steps, including the NMF factorization, clustering and label assignment, are fitted without noise. Synthetic samples are then drawn from the privatized summaries on a per-cluster, per-gene basis using the same sampling strategy as in the non-privatized NMF model. The P-NMF results presented throughout correspond to ∈=4 applied to the per-cluster gene means. The effect of varying budget $\in =\mathrm{1,4},\mathrm{10,50}$ on P-NMF is reported in Additional file 3: Fig. S22.

**Limitations:** In addition to the inherent linearity constraints of NMF, the Gaussian mechanism introduces a privacy–utility trade-off: stronger privacy (smaller ∈) requires more noise to be added to the released cluster means, reducing fidelity. Performance is also sensitive to the choice of NMF rank k, the public contribution bound *R* and to cluster sizes, since smaller clusters have higher sensitivity and therefore require larger noise.

#### 2. Probabilistic graphical models (PGM)

These methods learn an explicit probabilistic model over the variables using a dependency graph (e.g., Bayesian networks or Markov random fields). The joint distribution factorizes into local conditional or potential functions defined by the graph structure, and synthetic samples are generated by sampling from this fitted distribution [75]. We evaluated Differentially Private PGM as an instance of this family, which was submitted by the participants.

##### 2.1 Differentially private probabilistic graph model (DP-PGM)

Private-PGM [72] is a marginals-based differentially private synthetic data generator and uses the implementation provided in public repositories (https://github.com/ryan112358/mbi [76] and (https://github.com/MarieOestreich/PRO-GENE-GEN/blob/main/models/Private_PGM [77]). To distinguish formally DP from non-DP methods, we refer to it as DP-PGM throughout the manuscript. The method operates by first selecting a collection of low-dimensional marginals over the dataset, releasing them under differential privacy, and then fitting a graphical model that is consistent with the noisy marginals.

**Implementation:** For a given discretized dataset, (i) 1-way marginals for individual genes and (ii) 2-way marginals between each gene and class label were computed, yielding summary statistics that preserve feature-label dependencies. The privacy guarantee was defined at the record level using add/remove adjacency, i.e., where two neighboring datasets differ by the addition or removal of an individual’s complete record. The protected unit is, thus, the individual’s complete record. Each marginal *M*_*l*_ was perturbed using a DP mechanism to produce ${\widetilde{M}}_{l}$. Specifically, measurements were privatized using Gaussian noise, with the privacy budget accounted for using Rényi differential privacy (RDP), the privacy accounting combines marginal measurements ((i) and (ii)) to calibrate the mechanism to specified $(\in ,\delta )$-DP. A PGM was then estimated such that its implied marginals match $\{{M}_{l}\}$. The PGM inference and synthetic data generation operate on privatized marginals without additional privacy loss due to post-processing property. The resulting joint distribution factorizes over model cliques, and synthetic samples are obtained by sampling from this distribution.

Continuous gene-expression values were discretized using quantile binning into four equiprobable bins prior to marginal estimation. Model parameters were optimized using the default mirror-descent routine provided in the public repository. Four privacy-budget settings were evaluated $(\in =\mathrm{1,4},\mathrm{10,50})$ while all other hyperparameters were kept fixed.

**Limitations:** Fidelity depends on the chosen marginal set and discretization scheme; higher-order (involving three or more genes) or nonlinear gene–gene dependencies not captured by the factorization may be under-represented. In high-dimensional continuous settings, scalability and structural misspecification can limit biological correlation recovery. We also note that data discretization was performed without DP and was excluded from privacy accounting. Quantile thresholds were computed from the input dataset and were treated as precomputed, fixed inputs. The reported privacy budgets characterize marginal privatization and subsequent synthesis conditional on fixed discretization boundaries.

#### 3. Latent-variable autoencoders

The variational autoencoder (VAE) is a latent-variable generative model that consists of encoder-decoder neural architecture with a parametric latent prior, such as isotropic Gaussian [66]. The encoder maps each observation *x* to a distribution over latent variables *z*, while the decoder parametrizes the conditional likelihood $p\left(x\left|z\right.\right)$, enabling generation from the prior and decoding. VAEs are widely used to obtain low-dimensional, denoised representations that capture dominant sources of variation in RNA-seq data and related omics modalities. Following instances of the VAEs are evaluated in this benchmark:

##### 3.1 Conditional variational autoencoder (CVAE)

Conditional VAEs (CVAEs) augment the latent representation with sample-level covariates, allowing the generative process to respect experimental or biological attributes [65].

**Implementation:** The evaluated CVAE is implemented in Pytorch (2.4.0) [78] and employs a fully connected encoder-decoder architecture with three hidden layers per branch and ReLU activations. Conditioning variables (e.g., disease subtype/type) are incorporated either as one-hot vectors or via an embedding layer and are concatenated with both the encoder features and the latent representation in the decoder pathway. The latent space follows a Gaussian prior, and model training maximizes a \beta-weighted ELBO with mean-squared-error reconstruction loss and a KL-divergence regularizer, using the form $L=MSE+\beta \mathrm{K}\mathrm{L}$, with β=0.001. The comparative results for one-hot encoding and embedding layer based representation are included in the Additional file 2: Tables S4-S5.

**Limitations:** Like other autoencoder-based generators, CVAEs can exhibit reconstruction bias toward the training manifold, which may limit exploration of rare expression modes. Performance is sensitive to preprocessing choices (e.g. normalization) and to the relative weighting of the KL term, where excessive regularization may yield latent-space collapse while insufficient regularization can degrade sampling quality. Conditioning effectiveness depends on the representation of covariates (one-hot versus learned embeddings), and poorly tuned conditioning can lead to label leakage or reduced separation between biologically meaningful groups in the latent space. 

##### 3.2 Conditional variational autoencoder with Gaussian Mixture Models (CVAE-GMM)

The CVAE–GMM extends the baseline CVAE by replacing the standard isotropic Gaussian prior with a Gaussian mixture model (GMM) [79] in latent space, following the intuition of [68]. The objective is to better capture latent heterogeneity within a conditioning label, such as disease groups that may contain unobserved subtypes, by allowing multiple latent components rather than a single unimodal prior.

**Implementation:** The neural architecture and the training procedure are identical to the baseline CVAE. After training, the encoder is applied to the full (real) dataset to obtain posterior mean latent representations *z*. For each disease type or subtype label, relevant latent factors are selected and a GMM with ${n}_{\mathrm{c}\mathrm{o}\mathrm{m}\mathrm{p}\mathrm{o}\mathrm{n}\mathrm{e}\mathrm{n}\mathrm{t}\mathrm{s}}=3$ is fitted and stored for that group (in both benchmark datasets). During generation, latent samples are drawn from the corresponding fitted GMM rather than a standard normal distribution, and these samples are decoded using the trained CVAE decoder. Essentially, GMM acts as a dynamic data-adaptive mixture prior that replaces the single Gaussian prior used in the baseline CVAE.

**Limitations:** This approach introduces modeling complexity and requires careful selection of the number of mixture components, which may affect stability. Because GMM is fitted on the latent encodings derived from the original dataset, the method involves additional post-hoc pass over the data; which may increase the risk of latent overfitting and memorization, and therefore increase privacy vulnerabilities.

##### 3.3 Differentially private conditional variational autoencoder (DP-CVAE)

The DP-CVAE extends the baseline CVAE by incorporating a formal differential-privacy (DP) mechanism into training. The model architecture, encoder-decoder structure, and conditioning on the disease labels are identical to baseline CVAE, but per-sample gradients are clipped and perturbed with Gaussian noise to ensure privacy, providing $\in , \delta$ guarantees for the learned parameters.

**Implementation:** DP training was implemented with Opacus (v1.5.2) [80] which provides DP-SGD algorithm [2] in Pytorch. Therefore, DPCVAE adopts the record-level adjacency definition used by Opacus DP-SGD: two neighboring training datasets differ in the inclusion or exclusion of a single training example. The resulting ($\in ,\delta$)-DP guarantee protects the contribution of any individual training example to the released model. A privacy engine wraps the model, optimizer and data loader, and operates as follows:

1. Per-sample gradients are computed and clipped to a maximum *L*_2_ norm of 0.1.
2. Gaussian noise is added to the clipped gradients, with target privacy budget ∈, target $\delta ={10}^{-5}$, and batch size set to 64.
3. Privacy accounting is performed to track cumulative ∈ over all optimization steps.

To assess the privacy-utility trade-off we swept across $\in =\mathrm{1,4,10,50}$ (Additional file 3: Fig. S1).

**Limitation:** In addition to the baseline CVAE limitations, DP-CVAE introduces an inherent tradeoff between privacy and fidelity: stricter privacy budgets reduce reconstruction quality, latent-space separation, and preservation of biologically meaningful variation, while looser budgets improve utility but relax privacy guarantees. Model performance is sensitive to the choice of gradient clipping norm, noise scale, and batch size.

#### 4. Generative adversarial networks (GANs)

The generative adversarial networks are composed of two components, a generative model *G* that captures the data distribution, and a discriminative model *D* that estimates the probability that a sample came from the training data rather than *G*, both of which are trained simultaneously [81]. Training optimizes a min–max objective, effectively matching the generated distribution to the data distribution without requiring an explicit likelihood. Conditional variants allow generation to be guided by covariates (e.g., disease subtype), enabling targeted synthetic data creation. We evaluated the following instances in this benchmark.

##### 4.1 Wasserstein GAN with gradient penalty (WGAN-GP)

In Wasserstein GAN, the adversarial training follows the Wasserstein objective with gradient penalty to enforce a 1-Lipschitz constraint [69]. WGAN-GP replaces the standard GAN objective with a Wasserstein loss and gradient penalty to improve stability and global distribution matching [70].

**Implementation:** A conditional WGAN-GP where both the generator and discriminator are fully connected networks conditioned on disease subtype is trained. The generator maps latent Gaussian noise concatenated with a disease-condition vector to the expression feature space, using a multilayer feed-forward architecture with batch normalization, ReLU activations, and dropout. Gradient penalty set equal to λ=10 to enforce a 1-Lipschitz constraint, and for each generator update, the discriminator (critic) is updated ${n}_{\mathrm{c}\mathrm{r}\mathrm{i}\mathrm{t}\mathrm{i}\mathrm{c}}=5$ times, computing the loss using random linear interpolations between real and synthetic samples. The generator is optimized to maximize the critic's score on synthetic samples. Both networks are trained with Adam. Inputs are z-score normalized prior to training, and generated samples are inverse-transformed back to the original expression scale at synthesis time. Similar to CVAE, we investigated both one-hot disease encodings, and a learned embedding layer for compact condition representations (Additional file 2: Tables S4-S5).

**Limitations:** As with other GAN-based models, WGAN-GP may under-represent minority modes and is sensitive to critic-generator update balance and learning-rate tuning.

##### 4.2 Conditional tabular GAN (CTGAN)

CTGAN is a tabular focused GAN model with conditional sampling which handles imbalanced categories and mixed data types [67].

**Implementation:** The model uses a fully connected generator with stacked residual blocks and batch normalization, which maps a latent Gaussian input concatenated with a conditional vector to the transformed feature space. The discriminator operates on PAC-grouped samples (pac = 10), enforcing a 1-Lipschitz constraint via a Wasserstein loss with gradient penalty (WGAN-GP). Conditioning is implemented by concatenating the one-hot-encoded label vector to both the generator and discriminator inputs, supporting label-guided sample synthesis.

Training follows the reference CTGAN implementation, using mode-specific data normalization. The generator is optimized with Adam, and the model is trained 10,000 iterations with a batch size of 64. Synthetic samples are generated by sampling latent noise and conditioning vectors. Synthetic samples are produced by sampling latent noise and conditioning vectors at generation time.

**Limitations:** CTGAN inherits known GAN trade-offs, including mode under-coverage though it attempts to solve this via the mode-specific normalization. Performance is sensitive to the balance between discriminator and generator learning rates. Preprocessing may distort marginal RNA-seq feature structure, and adversarial optimization remains less stable than likelihood-based approaches.

##### 4.3 Differentially private conditional tabular GAN (DP-CTGAN)

DP-CTGAN extends the baseline CTGAN implementation by incorporating differential privacy.

**Implementation:** DP training was implemented with Opacus (v1.5.2) via the DP-SGD algorithm in PyTorch and SmartNoise-Synth (https://docs.smartnoise.org/). Record-level adjacency, where two neighboring datasets differ in one complete training record is assumed. Thus, the protected unit is an individual training record (i.e. patient), including both its feature values and associated label. The preprocessing step and the subsequent training of the synthesizer are both performed under an ($\in ,\delta$)-DP guarantee. Privacy loss, therefore, can arise from the differentially private preprocessing and from DP-SGD used to train DPCTGAN. Per-sample gradients are *l*2-clipped and perturbed with Gaussian noise; the generator is trained normally using the discriminator’s privatized outputs. Privacy budgets are accounted for using the moments accountant, with target $\delta=10^{-5}$, batch size set to 64 and per-sample gradient clipping set to 0.1. A preprocessor epsilon is also applied to compute bounds for each feature, scaled by preprocessor-eps-multiplier = 0.1. For high-dimensional data (978 features), this preprocessor step consumes a significant portion of the privacy budget, which limits the minimum achievable epsilon. In our experiments, training with ∈<45 was unstable or failed, whereas ∈=50 successfully ran, reflecting the combined cost of generator training and preprocessing.

**Limitations:** In addition to baseline CTGAN limitations, differential privacy introduces a strict privacy-utility tradeoff, where high-dimensional data amplifies privacy cost. The preprocessor’s contribution to epsilon reduces flexibility in achieving stricter privacy guarantees. As with most DP-GAN approaches, only the discriminator is privatized; the generator receives gradients through the privatized discriminator, so overall privacy guarantees rely on correct accounting of DP-SGD in the discriminator.

#### 5. Diffusion models

A diffusion model consists of two key phases: a forward diffusion stage, where input data is gradually perturbed with Gaussian noise over multiple steps, and a reverse diffusion stage, in which the model systematically reconstructs the original data by reversing this process. Originally introduced in 2015 [82], and later popularized through denoising diffusion probabilistic models [83], and score-based generative models [84], diffusion models have shown strong performance across high-dimensional and complex data, including images, text, and tabular data [64]. In this benchmark, we evaluated the following instance of a diffusion model:

##### 5.1 Embedded noisy diffusion

**Implementation:** The core strategy of the Embedded Noisy Diffusion is to use a conditional diffusion model with DP-SGD [71]. Timesteps are encoded via sinusoidal positional embeddings and class labels via neural embeddings, combined into a single conditioning vector. The network comprises ResLinear residual blocks with Group Normalization (8 groups), SiLU activations, dropout (0.2), and a progressive dimensionality scheme for embedding bottlenecks. Differential privacy is applied through DP-SGD with gradient clipping (max-grad-norm = 1.0) and a minimal noise multiplier (dp-noise-multiplier = 0.00001), chosen to preserve accuracy while providing privacy guidance. SMOTE oversampling [85] is adopted to balance class representation, and features are scaled with a QuantileTransformer and inversely transformed to maintain biological interpretability. The denoising objective is optimized with MSE loss, AdamW, a OneCycleLR scheduler, and early stopping.

**Limitations:** While DP-SGD is applied, the extremely low noise multiplier (0.00001) means the model cannot provide a formal privacy guarantee, therefore this model is not considered as a DP model in this benchmark. Additionally, the model requires careful tuning of ResLinear blocks and normalization for stability, and training remains computationally intensive despite inference being efficient.

### Evaluation framework

All models were evaluated under identical data splits, random seed, and preprocessing pipelines to ensure comparability. All synthetic datasets were generated with identical sample size and feature dimensionality as the training dataset (per each fold). Resource consumption for training and data generation per generative model across both datasets is reported in Additional file 2: Tables S6-S7. Because the evaluated models were executed on different hardware configurations, the reported times and memory are provided together with the corresponding hardware specifications for reference and should not be interpreted as directly comparable across models.

We evaluate models along four complementary dimensions: (i) statistical fidelity to the real data distribution, (ii) downstream task utility, (iii) biological plausibility of generated features, and (iv) privacy risk. Additional file 2: Table S1 presents the complete set of metrics that are used in evaluation.

#### Fidelity

Fidelity evaluates how closely the synthetic data matches the statistical properties of the real data. We quantify distributional similarity using Maximum Mean Discrepancy (MMD) and gene-wise Kullback–Leibler (KL) divergence.

MMD was computed using an RBF kernel with bandwidth parameter γ=1/d where *d* denotes the number of features, and is evaluated between synthetic samples and the real training set (MMD-train), as well as between synthetic samples and the real test set (MMD-test). Lower MMD (close to 0) indicates closer alignment between real and synthetic distributions.

KL divergence was computed across marginal (gene-wise) feature distributions in the direction $\mathrm{K}\mathrm{L}\left({P}_{\mathrm{r}\mathrm{e}\mathrm{a}\mathrm{l}}\Vert {P}_{\mathrm{s}\mathrm{y}\mathrm{n}}\right)$, treating the real data distribution as reference. Marginal densities were estimated using fixed-width histogram binning with shared bin edges and ∈-smoothing (10^–10^) to avoid numerical instability. KL values were averaged across genes. We report synthetic-vs-real-train (authenticity) and synthetic-vs-real-test (generalizability) values. Lower KL divergence (close to 0) reflects better preservation of feature-level statistical structure.

We compute density and coverage metrics [38] to evaluate the similarity between the manifolds of real and synthetic data. Both metrics are based on local neighborhoods defined by k-nearest neighbors of each real data sample, with *k* = 5. For each synthetic sample, density measures how many real-sample neighborhoods it falls within (normalized by k), providing an estimate of local fidelity. A density value near 1 indicates synthetic local density matching real, values above 1 indicate concentration in high-density regions, and it is interpreted jointly with coverage. Coverage, on the other hand, measures the fraction of real samples whose neighborhood contains at least one synthetic sample, to measure diversity. Coverage is bounded between 0 and 1, and higher value indicates better diversity. We use the publicly available implementation provided by the authors (https://github.com/clovaai/generative-evaluation-prdc).

We compute the distance-to-closest record statistic between synthetic and real (train) samples as a measure of local coverage of the real data and to identify potential oversampling or sample memorization. Smaller distances indicate closer alignment between synthetic and real samples. The distance-to-closest metric was reported relative to the base value computed as the distance-to-closest within real train and real test data. While distance-to-closest is informative for fidelity, it is also commonly interpreted in the context of privacy; we therefore revisit this metric in the privacy evaluation section.

Finally, to assess whether synthetic samples are statistically distinguishable from real data, we compute a discriminative score using a logistic regression classifier trained to separate real training samples from synthetic samples. The combined dataset (real train + synthetic) is randomly split into training and test partitions. After training, evaluation is performed on an extended test set that includes (i) the held-out portion of the mixed real/synthetic data and (ii) independent real test samples not used during classifier training. Performance is quantified using the F1 score and referred to as the *discriminative score*, where higher values indicate easier separation between real and synthetic samples.

#### Utility

We evaluate downstream task utility by measuring how well models trained on synthetic data generalize to real data in relevant predictive settings. For TCGA-BRCA, the downstream task is molecular subtype prediction; for TCGA-COMBINED, the downstream task is multi-class cancer type prediction across multiple tissues. Throughout the evaluation, “downstream applications” refer specifically to these tasks, which also serve as proxies for biological utility at different levels of granularity.

We train a one-vs-rest Logistic Regression classifier on the synthetic training set and evaluate its performance on the held-out real test set (train-on-synthetic, test-on-real, TSTR). As a reference, we train the same classifier on the real training set and evaluate it on the same real test set (train-on-real, test-on-real, TRTR). Evaluation is performed under five-fold cross-validation, and mean performance across folds is reported. To account for class imbalance, we report multiple evaluation metrics, including accuracy, macro- and class-weighted precision–recall AUC, F1 score, and AUC-ROC. In the main text, we focus on AUC-ROC and F1 score as the primary metrics; the remaining metrics are available through the public Github repository. Alignment between synthetic-trained and real-trained performance indicates preservation of task-relevant structure. For interpretability, we additionally report relative scores (e.g., AUC-ROC relative, F1 relative), defined as the performance of the models trained on synthetic data normalized by the performance achieved when trained on real data (perf_synthetic_/perf_real_). Relative values closer to one indicate stronger downstream utility of the synthetic data.

To assess whether the synthetic data preserves feature-level signals, we additionally compute the count and percentage (%) of overlapping predictive genes identified in the synthetic-trained model that are also among the top predictive genes in the real-trained model. For each class, we extract the top-10 features corresponding to the largest positive Logistic Regression coefficients from the one-vs-rest classifiers, aggregate these per-class feature sets, and compare their overlap between synthetic-trained and real-trained models. Higher overlap indicates stronger retention of biologically and task-relevant structure in the synthetic data.

#### Biological plausibility

Unlike other domains where synthetic data generation is applied, healthcare data, especially high-dimensional genomics data, requires additional evaluation metrics to ensure validity and reliability. For bulk RNA-seq datasets, assessing biological plausibility is critical, including the ability of synthetic data to reproduce meaningful differential expression patterns (e.g., between molecular subtypes or cancer types) and realistic gene co-expression structures. We followed a biological utility analysis workflow similar to [22], using their provided codebase (https://github.com/MarieOestreich/PRO-GENE-GEN/).

##### Gene co-expression

We employed the hCoCena (horizontal construction of co-expression networks and analysis), an R-package for network-based joint co-expression analysis [39], to quantify co-expression preservation between the synthetic and real datasets. In these networks, nodes represent genes and edges represent co-expression relationships weighted by Pearson correlation coefficient (*r*). Edges below a user-defined correlation cutoff can be discarded, allowing focus on co-expression relationships across varying strengths. Analyses were performed for each fold, with correlation thresholds of *r* = 0.0, *r* = 0.3 and *r* = 0.5 to evaluate network recovery across datasets. We then quantified co-expression recovery using three complementary metrics. First, the True Positive Rate (TPR) measures the fraction of real co-expression edges correctly recovered in synthetic network: $\mathrm{T}\mathrm{P}\mathrm{R}=\frac{\mathrm{n}\mathrm{u}\mathrm{m}\mathrm{b}\mathrm{e}\mathrm{r}\;\mathrm{o}\mathrm{f}\;\mathrm{c}\mathrm{o}\mathrm{r}\mathrm{r}\mathrm{e}\mathrm{c}\mathrm{t}\;\mathrm{e}\mathrm{d}\mathrm{g}\mathrm{e}\mathrm{s}}{\mathrm{n}\mathrm{u}\mathrm{m}\mathrm{b}\mathrm{e}\mathrm{r}\;\mathrm{o}\mathrm{f}\;\mathrm{r}\mathrm{e}\mathrm{a}\mathrm{l}\;\mathrm{e}\mathrm{d}\mathrm{g}\mathrm{e}\mathrm{s}}$. Second, the false edge rate measured the number of spurious edges (false correlations) introduced by the synthetic network relative to the real network: $\mathrm{F}\mathrm{a}\mathrm{l}\mathrm{s}\mathrm{e}\;\mathrm{e}\mathrm{d}\mathrm{g}\mathrm{e} \;\mathrm{r}\mathrm{a}\mathrm{t}\mathrm{e}=\frac{\mathrm{n}\mathrm{u}\mathrm{m}\mathrm{b}\mathrm{e}\mathrm{r} \;\mathrm{o}\mathrm{f} \;\mathrm{f}\mathrm{a}\mathrm{l}\mathrm{s}\mathrm{e}\;\mathrm{e}\mathrm{d}\mathrm{g}\mathrm{e}\mathrm{s}}{\mathrm{n}\mathrm{u}\mathrm{m}\mathrm{b}\mathrm{e}\mathrm{r} \;\mathrm{o}\mathrm{f}\; \mathrm{r}\mathrm{e}\mathrm{a}\mathrm{l}\; \mathrm{e}\mathrm{d}\mathrm{g}\mathrm{e}\mathrm{s}}$. Finally, we defined a co-expression specificity score to summarize network fidelity $\mathrm{S}\mathrm{p}\mathrm{e}\mathrm{c}\mathrm{i}\mathrm{f}\mathrm{i}\mathrm{c}\mathrm{i}\mathrm{t}\mathrm{y}=\frac{1}{1 + \mathrm{f}\mathrm{a}\mathrm{l}\mathrm{s}\mathrm{e} \;\mathrm{e}\mathrm{d}\mathrm{g}\mathrm{e}\; \mathrm{r}\mathrm{a}\mathrm{t}\mathrm{e}}$ where higher values indicate fewer spurious edges per true edges. Together, higher TPR and higher specificity indicate that the synthetic dataset preserves the real co-expression structure while minimizing falsely introduced correlations.

##### Differential expression

Differential expression (DE) between molecular subtypes (TCGA-BRCA) or cancer types (TCGA-COMBINED) was evaluated for all pairwise class comparisons using the nonparametric Wilcoxon test implemented in the scran R package [86]. This yielded 10 pairwise molecular subtype comparisons in TCGA-BRCA (e.g. HER2-LumA) and 66 pairwise cancer type comparisons in TCGA COMBINED (e.g. Skin-Colorectal) dataset. DE analysis was performed in the same VST-transformed space for both real and synthetic datasets.

Up- and down-regulated genes were analyzed separately for each comparison. Genes were considered differentially expressed (DE) if they met both a statistical significance threshold (adjusted *p* ≤ 0.05, Benjamini–Hochberg FDR correction with (α=0.05) and a minimum effect-size threshold (ΔVST), implemented via the lfc argument in pairwise Wilcoxon function in scran R-package on VST-transformed expression values. The effect-size threshold corresponds to the minimum difference in median VST expression between groups required for a gene to be called DE. We assessed the effect-size thresholds at 0.0, 0.2, 0.5, and 1.0 across both datasets to assess both sensitivity and potential shifts (Additional file 3: Fig. S6).

True Positive Rate (TPR) was defined as the proportion of real DE genes correctly identified in the synthetic data, while False Positive Rate (FPR) was the proportion of non-DE genes incorrectly called as DE. Metrics were computed separately for upregulated and downregulated genes.

##### Pathway analysis

Pathway recovery was evaluated with single-sample GSEA (ssGSEA) using Gene Set Variation Analysis (GSVA) R package [40]. Enrichment scores were computed for the 50 molecular signature database (MSigDB) Hallmark gene sets [41, 87] in both real and synthetic datasets to assess how well each generative model preserved biologically meaningful pathway activity compared to real data.

For each pathway, real and synthetic score distributions (pooled across the cohort) were compared with a two-sample Kolmogorov–Smirnov (KS) test, and a signed standardized effect size (Cohen's d), computed as the mean difference (synthetic − real) divided by the pooled standard deviation $\left(d=\frac{{\overline{x} }_{syn}-{\overline{x} }_{real}}{{S}_{pooled}}\right)$. KS test *p*-values were corrected for multiple testing across pathways within each fold using the Benjamini–Hochberg false discovery rate (FDR) procedure. The fraction of pathways showing a significant distributional shift was then averaged across folds. Pathway-level dispersion was summarized as the geometric mean of the per-pathway variance ratios (synthetic/real), since variance ratios are symmetric in log space.

#### Privacy

##### Threat model

All privacy evaluations are conducted under a black-box adversarial setting with no query access to the generator. The adversary is assumed to have access only to the released synthetic dataset and a set of candidate records, comprising both members (real training samples) and non-members (held-out real test samples), but does not know which candidates were used in training. The adversary has no access to generative model parameters, gradients, or internal representations.

All data, including synthetic outputs, membership test candidates, and reference cohorts, are represented in variance-stabilizing transformation (VST) space, consistent with the representation used throughout fidelity and utility assessments and reflecting the realistic post-release scenario in which a data recipient receives synthetic data in the same processed form used for downstream analyses. Candidate records correspond to the same physical samples as the training set; no biological replicates were used. This ensures a well posed membership test that is (i) unambiguous, since each candidate either was or was not in the training set, and (ii) conservative, since testing exact training records gives the adversary the strongest possible membership signal.

For reference-anchored attacks, the adversary is additionally granted a disjoint reference cohort withheld from the original dataset prior to all model training and evaluation, representing auxiliary population-level knowledge rather than privileged training information.

This black-box, output-only threat model (also referred to as no-box and no-box calibrated in some parts of literature) is consistent with the standard adopted in the synthetic data privacy literature [43, 88]. White-box and model-access attacks, while theoretically stronger, can be avoided by not publishing the generative model itself and makes the output-only setting the operationally relevant attack surface for a cautious data publisher [43, 88]. Furthermore, though the data publisher controls sharing of their own data, they cannot control whether an adversary independently acquires population-level data from the general population [43, 88]. The inclusion of reference-anchored attacks therefore represents a realistic and conservative upper bound on privacy risk under this threat model.

##### Membership inference attacks

We adopted a suite of complementary membership inference attack (MIA) methods to assess whether the generative models exhibit memorization or disproportionate influence from individual training samples. We implemented these attacks using the DOMIAS package (https://github.com/holarissun/DOMIAS), extending it where necessary for our setting.

**Attack categories:** we evaluate three categories of attacks.

*Distance-based attacks* (Monte Carlo (MC) [42], GAN-leaks [37] and GAN-leaks calibrated [37]) operate directly in the data space and quantify whether a candidate sample lies unusually close to the synthetic samples generated by the model. GAN-leaks scores each candidate by its minimum squared distance to the synthetic dataset, exponentiated with median-based distance scaling to avoid numerical underflow in high-dimensional space, while MC scores each candidate by the fraction of synthetic samples falling within an adaptive nearest-neighbor distance threshold. GAN-leaks calibrated extends GAN-leaks by additionally referencing each candidate's minimum distance to a held-out reference cohort, contrasting closeness to synthetic against closeness to reference.

The *density-based attack (*DOMIAS-KDE [43]) estimates a density ratio between the synthetic and reference dataset distributions using a kernel density estimator, with the score reflecting regions where the generator disproportionately oversamples training-like signals.

*Classifier-based attacks* [89] (Confidence-LR, Confidence-RF) train downstream classifiers (logistic regression and random forest) on the synthetic data and use the maximum predictive class probability per candidate as a membership score. This attack probes whether models trained on synthetic data retain systematically higher confidence on records resembling training members relative to non-members. LOGAN-D1 [44] instead, trains a discriminator to distinguish synthetic from reference samples and scores candidates by its output.

As explained in their descriptions, three of these attacks (GAN-leaks calibrated [37], LOGAN-D1 [44], and DOMIAS-KDE [43]) are reference-anchored, indicating that they additionally contrast candidate records against a held-out reference cohort (e.g. an auxiliary dataset) to disentangle training-specific memorization signals from general population structure. More details on each attack model can be found in Additional file 2: Table S2.

The Monte Carlo attack is reported descriptively but excluded from cross-model comparisons, as its per-model median-distance calibration (the median heuristic) makes its scores non-comparable across generators.

**Feature selection for reference-anchored attacks:** Three reference anchored attacks, GAN-leaks calibrated, LOGAN-D1, DOMIAS-KDE, compare candidate records against both synthetic and reference distributions. Of these, only DOMIAS-KDE structurally requires dimensionality reduction, as kernel density degrades in high dimension, while GAN-leaks calibrated and LOGAN-D1 operate natively on full data. To enable a controlled comparison across reference-anchored attacks, we evaluated these methods under four feature representations, each retaining the top 100 dimensions: (i) *principal component analysis* (*PCA)* where principal components fitted on the reference set and applied to test and synthetic data; (ii) *differentially variable genes (DVG)* selected by synthetic-to-reference variance ratio; (iii) *supervised differentially expressed genes (SDEG)* selected by mutual information with cancer type and subtype labels derived from the synthetic release; and (iv) *discriminatively selected genes (DG)* identified by an L1-regularised logistic regression trained to separate synthetic from reference samples. In all cases, feature selection and dimensionality reduction were computed using only the reference set and synthetic data to prevent leakage of membership information into the attack pipeline. Each representation is also attack-agnostic where features are selected once and applied identically to all three attacks, so the column-wise comparison reflects differences in attack mechanism rather than representation. This yielded four attack configurations per reference-anchored method, enabling assessment of privacy risk robustness across different feature representations. More details on each feature representation method can be found in the Additional file 2: Table S3.

**Reference dataset:** A single reference cohort was constructed by withholding 824 samples from the TCGA-COMBINED dataset prior to all model training and evaluation. This reference set was used exclusively for TCGA-COMBINED relevant attacks, where the reference population is well-matched to the training distribution. For TCGA-BRCA, the limited cohort size precluded construction of a matched reference cohort without substantially reducing training data; and therefore, reference-anchored attacks were therefore not applied to this dataset.

**Dataset-specific attack protocols:** Distance-based and classifier-based attacks were applied to both TCGA-BRCA and TCGA-COMBINED.Reference-anchored attacks, including density-based DOMIAS-KDE, were applied to TCGA-COMBINED only,using a reference dataset across all four feature representations.

**Experiment setup:** The real dataset served as the membership query set and was evaluated against the corresponding synthetic data generated by each model. Membership inference risk was assessed separately for each fold under five-fold cross-validation. Since 80% of the data are used for training in each fold, the positive (member) class prevalence is 0.8.

##### Metrics

Each attack returns a continuous membership probability, where higher values indicate a greater likelihood of membership. For each generator, we report the worst case across attacks (i.e. the strongest-performing attack per model) as a conservative estimate of privacy risk, so that a generator is considered vulnerable if any evaluated attack succeeds. We report attack success using AUC-ROC, a standard threshold-independent measure, together with the true positive rate (TPR) at a fixed false positive rate (FPR = 0.1). AUC-ROC is prevalence-independent and therefore measures whether an attack ranks training members above non-members, rather than an absolute disclosure probability, which would additionally depend on the membership prior. AUC-ROC also provides an average-case summary across thresholds that may obscure high-risk cases where specific individuals are particularly exposed. The TPR at a low fixed FPR partially addresses this by quantifying attacker success in the high-confidence regime. For example, the fraction of true members correctly identified when only 10% of non-members are misclassified. While TPR@FPR does not fully characterize per-individual exposure, it provides visibility into low FPR regions, which an average summary can otherwise miss.

Because the membership test set comprises the full dataset while only 20% of samples are non-members under five-fold cross-validation, the resulting class imbalance can bias threshold-dependent metrics. A precision-recall-based metric, AUC-PR, computed under this class balance (with its prevalence-determined random-classifier baseline) is also reported in the Additional file 3: Figs. S11 and S14, for both datasets, respectively.

In addition to attack-based metrics, similarity-based measures are sometimes used to estimate privacy risk. The distance to the nearest real neighbor (distance-to-closest) computes the average distance from each synthetic point to its closest real counterpart. GAN-Leaks is also based on nearest-neighbor distance but differs in that it is computed per candidate record in the membership test set, yielding a membership score rather than a dataset-level average. The relationship between the attack-based metrics and distance-to-closest is examined in Additional file 3: Fig. S18.

##### Operational interpretation of the threat model

The presented threat model corresponds to a data publisher releasing a synthetic dataset while retaining the generative model. The adversary is a recipient holding candidate records who seeks to determine whether specific individuals were in the training data which is a sensitive disclosure, since training membership here implies inclusion in a cancer cohort. The best-performing membership inference attack performance we report is the quantity a cautious publisher would consult before release. A near-baseline performance (TPR@FPR = 0.1≈0.1; AUC-ROC≈0.5) indicates low membership-disclosure risk, while elevated performance flags a generator warranting mitigation. As noted in the Limitations, this interpretation holds under our pipeline assumptions (shared representation, pre-processing), and realistic deployments with measurement or normalization mismatch would modify attainable MIA performance, so the reported values are a pipeline-dependent estimate rather than an absolute deployment risk.

#### Correlation analyses across evaluation metrics

We assessed pairwise correlations between evaluation metrics using model-level summary results. For each generative method, performance was averaged across five cross-validation folds prior to analysis, yielding one aggregate value per metric per method. Consequently, the effective sample size for correlation analysis was *n*=11, corresponding to the number of evaluated methods.

Spearman’s rank correlation (ρ) was used to quantify monotonic associations between metrics. Statistical significance was evaluated using two-sided tests, and *p*-values were adjusted for multiple comparisons using the Benjamini–Hochberg procedure to control the false discovery rate (FDR) at α=0.05. Adjusted *p*-values (q-values) are reported, with significance thresholds denoted as, * = *q* < 0.05, ** = *q* < 0.01, and *** = *q* < 0.001.

Two correlation panels were computed: (i) Fidelity and downstream utility metrics (13 metrics in total), presented in Additional file 3: Figs. S2-S3 and (ii) cross evaluation-axis integration, including 11 representative metrics spanning all four evaluation axes (fidelity, utility, biological plausibility, and privacy), shown in Additional file 3: Figs. S19-S20.

## Supplementary Information

Additional file 1: Challenge description.

Additional file 2: Supplementary tables.

Additional file 3: Supplementary figures.

## Data Availability

Datasets Datasets We used open access bulk gene-expression data from The Cancer Genome Atlas (TCGA), accessed via the Genomic Data Commons (GDC) portal (https://portal.gdc.cancer.gov) (Methods). The preprocessed datasets generated, analyzed and supporting the conclusions of this article are publicly available at the ELSA Benchmark platform [34] and at Zenodo (https://doi.org/10.5281/zenodo.22996320) [91]. Use of the data is subject to the NIH Genomic Data Sharing Policy and TCGA citation guidelines. Methods The baseline and post-challenge methods as well as all evaluation metrics are available in the challenge Github repository (https://github.com/PMBio/Health-Privacy-Challenge [92] and Zenodo (https://doi.org/10.5281/zenodo.23001599) [93]) under BSD-3-Clause license. The code is written for Python 3.10.14 and R 4.3.2 on Linux/Unix. The participant submitted methods are publicly available in both Github and Zenodo, compliant with the challenge format: Embedded Diffusion: https://github.com/not-a-feature/CAMDA25_NoisyDiffusion [94] and https://doi.org/10.5281/zenodo.22693680 [95]; NMF/P-NMF: https://github.com/AndrewJWicks/BulkNMFGenerator [96] and https://doi.org/10.5281/zenodo.22818979 [97]; DP-PGM: https://github.com/sikhapentyala/Health-Privacy-Challenge/tree/main/submission [98] and https://doi.org/10.5281/zenodo.23068244 [99]. Details of the original challenge, including the leaderboard and submissions, are hosted on the ELSA Benchmark platform [34].
