# Supplementary material for: Community benchmarking of generative models for privacy-preserving synthetic bulk RNA-seq data generation
Source: Genome Biol. 2026 Nov 9;27:355. doi: 10.1186/s13059-026-04326-1 (PMC13756914; doi:10.1186/s13059-026-04326-1)
Supplement: Supplementary file 1 [file 13059_2026_4326_MOESM1_ESM.docx]

Community benchmarking of generative models for privacy-preserving synthetic bulk RNA-seq data generation

Hakime Öztürk^*,1,2^, Tejumade Afonja^3^, Joonas Jälkö^4^, Ruta Binkyte^3^, Pablo Rodriguez-Mier^5^, Sebastian Lobentanzer^6^, Andrew Wicks^◇,7,8^, Jules Kreuer^◇,9^, Sofiane Ouaari^◇,9^, Nico Pfeifer^◇,9^, Shane Menzies^◇,10^, Sikha Pentyala^◇,10^, Daniil Filienko^◇,10^, Steven Golob^◇,10^, Patrick McKeever^◇,10^, Jineta Banerjee^◇,11^, Luca Foschini^◇,11^, Martine De Cock^◇,10,12^, Julio Saez-Rodriguez^5,13,14^, Mario Fritz^3^, Oliver Stegle^*,1,2^, Antti Honkela^*,4^

* : Corresponding authors ([hakime.oeztuerk@embl.de](mailto:hakime.oeztuerk@embl.de); [oliver.stegle@embl.de](mailto:oliver.stegle@embl.de); [antti.honkela@helsinki.fi](mailto:antti.honkela@helsinki.fi))

◇: Challenge participants that contributed method, synthetic data and CAMDA abstracts.

## Affiliations

**^1^European Molecular Biology Laboratory (EMBL), Genome Biology Unit, Heidelberg, Germany**

**^2^Division of Computational Genomics and Systems Genetics, German Cancer Research Center (DKFZ), Heidelberg, Germany**

**^3^CISPA Helmholtz Center for Information Security, Saarbrücken, Germany**

^4^**University of Helsinki, Helsinki, Finland**

^5^**Institute for Computational Biomedicine, Faculty of Medicine, Heidelberg University, Heidelberg, Germany**

^6^**Helmholtz Munich, Munich, Germany**

^7^**Division of Tumorigenesis and Molecular Cancer Prevention, German Cancer Research Center (DKFZ), Heidelberg, Germany**

^8^**DKFZ Hector Cancer Institute at the University Medical Center Mannheim, Mannheim, Germany**

^9^**Eberhard Karls Universität Tübingen, Tübingen, Germany**

^10^**University of Washington Tacoma, Washington, USA**

^11^**Sage Bionetworks, Seattle, USA**

^12^**Ghent University, Ghent, Belgium**

^13^**European Molecular Biology Laboratory, European Bioinformatics Institute (EMBL-EBI), Hinxton, UK**

^14^**Heidelberg University Hospital, Heidelberg, Germany**

# Supplementary Materials

## Challenge infrastructure

The Health Privacy Challenge was launched in conjunction with the annual international Critical Assessment of Massive Data Analysis (CAMDA) 2025 conference (<https://bipress.boku.ac.at/camda2025/>), which runs together with the Intelligent Systems for Molecular Biology (ISMB) conference series.

The challenge comprised two tracks: Track-1 focused on synthetic and private bulk RNA-seq generation, re-distributing two datasets obtained from the GDC portal ([https://portal.gdc.cancer.gov](https://portal.gdc.cancer.gov/analysis_page?app=Downloads)) containing The Cancer Genome Atlas (TCGA) [^1^](https://sciwheel.com/work/citation?ids=48935&pre=&suf=&sa=0) cohorts. Track 2, on the other hand, focused on synthetic single-cell RNA generation and re-distributed raw counts of OneK1K single-cell RNA-seq dataset (<https://onek1k.org/>), a cohort containing 1.26 million peripheral blood mononuclear cells (PBMCs) of 14 cell types from 981 donors[^2^](https://sciwheel.com/work/citation?ids=12772994&pre=&suf=&sa=0). The right for the distribution was kindly granted by the authors. To be considered valid, a submission had to include both a method submitted on the ELSA Benchmarks Platform ([https://benchmarks.elsa-ai.eu/?ch=4](https://benchmarks.elsa-ai.eu/?ch=4&com=introduction) [^3^](https://sciwheel.com/work/citation?ids=19179045&pre=&suf=&sa=0) ) and an accompanying extended abstract submitted to CAMDA 2025. Submissions that did not meet both requirements were not considered valid and were not evaluated.

## Track 1 (Bulk RNA-seq) - Leaderboard format

Track 1 was structured as a “Blue Team vs. Red Team” challenge, and implemented in two sequential phases to ensure that Red Teams had a sufficiently large and diverse set of Blue Team submissions to engage with.

### Blue Team setup

During the first phase (Phase I), Blue Teams developed generative models aimed at improving upon the provided baseline approaches and advancing methodological insight into privacy preservation for biological datasets.

The participants were provided with the re-distributed TCGA bulk RNA-seq datasets, denoted as $D$. Each team trained a generative model $f(D)$ to produce synthetic data derived from the original dataset. To prevent information leakage and enforce methodological independence:

- Each Blue Team selected a private, team-specific random seed, disclosed only to the organizers at submission,
- The seed determined the train-test split of $D$.
- The same split was used to train the generative model $f(D)$.

**Cross-validation protocol:** A five-fold cross-validation setting was utilized, where shape-matched synthetic datasets ${D_{SYN}}^{(i)}$are generated from the model $f(D)$ trained on the training set ${D_{TR}}^{(i)}$of each fold $i\in\{1,2,3,4,5\}$. The quality of the synthetic datasets were evaluated on the corresponding held-out test set ${D_{TE}}^{(i)}.$Performance metrics were averaged across folds to obtain a stable and generalizable estimate of the model performance.

**Membership Inference Setup:** For each cross-validation fold, a dedicated membership inference test dataset was constructed for subsequent use by Red Teams. Across folds:

- Members corresponded to samples included in the training partition.
- Non-members corresponded to samples in the held-out test partition.
- Membership composition varied by fold.

As five-fold cross-validation was employed, a total of five membership inference datasets were generated per Blue Team submission. By design, members constituted 80% of each inference dataset, reflecting 80/20 train test split.

Because each Blue Team used a distinct random seed, membership labels differed across teams. This avoided a fixed global split and introduced variability essential for robust Red Team evaluation.

**Timeline:** Blue Teams were only permitted to submit their methods during the Phase I of Track 1, which ran from January 14th 2025 to March 15th 2025.

### Red Team Setup

Track 1 incorporated the Red Team component in two sequential phases aligned with the Blue Team workflow.

**Phase I — Preparation and baseline attacks:** During the first phase of Track 1, Red Teams ran membership inference attacks (MIA) against synthetic datasets generated by the organizer-provided baseline generative models, using the baseline attack methods supplied by the organizers. These baseline attacks, not any participant-developed attack, correspond to the privacy evaluation reported in the main text, and ran in a black-box, output-only setting (see Threat Model, Methods): all attacks operate solely on released synthetic data (and, for reference-anchored attacks, an auxiliary reference cohort), without access to model parameters, gradients, or internal representations. This phase served two purposes: (i) to identify Red Teams intending to participate in Phase II, and (ii) to let participants develop, calibrate, and validate their MIA pipelines before evaluating Blue Team submissions.

**Phase II — Evaluation of Blue Team submissions:** In the second phase, Red Teams would launch MIA on the synthetic datasets and corresponding white-box generative models submitted by Blue Team participants. As no Red Team submission met the validity requirements (see above), this phase was not formally evaluated. We describe the intended design below for reference.

Here, white-box access indicates that Red Teams would be provided full visibility into model architecture, training procedure, and hyperparameters. The only withheld elements were the random seed for the train–test partition and the seed governing model initialization and training randomness (which may in some cases have coincided); neither was disclosed to Red Teams.

- Four Blue Team submissions were selected based on leaderboard performance on the benchmark platform, and shared with the Red Teams.
- Each Red Team was required to launch their MIA algorithm against at least one Blue Team model.
- If a Red Team did not explicitly attack a given Blue Team model, that model was evaluated using a predefined baseline MIA algorithm based on a Monte Carlo (MC) strategy [^4^](https://sciwheel.com/work/citation?ids=11211853&pre=&suf=&sa=0).
- If a team participated in both roles (Blue and Red), and their own Blue Team model was among the selected submissions, baseline MC performance was used in place of a self-attack to avoid conflicts of interest.
- Final Red Team attack performance was computed as the average performance across all evaluated Blue Team models, combining participant-submitted MIAs where provided and baseline MC evaluations where attacks were not conducted.

The white-box access described here applied only to the Track 1 Red Team (attack development). As no Red Team submission met the validity requirements, no participant-developed white-box attacks were evaluated, and the privacy results in the main manuscript use only the Phase I black-box baseline attacks.

**Timeline:** Phase II ran from March 22, 2025 to May 12, 2025.

### Evaluation

For Track 1, the submission platform executed a Docker container in the background, prepared and maintained by the organizers, to ensure a standardized and reproducible evaluation. Upon submission, the container automatically evaluated provided synthetic datasets using the predefined metrics specified in the official challenge Github repository. Each submission was required to include:

- A short methodological description,
- Python source files implementing the method,
- A configuration file specifying the hyperparameters used for training and generation, and
- An environment specification file to ensure computational reproducibility.

The resulting performance metrics were then published on the public leaderboard, making them visible to participants in both the Blue Team and Red Team tracks, as well as external observers interested in the challenge outcomes. This setup ensured transparency and consistency across submissions.

## Track 2 (Single-cell RNA-seq) - Open research format

Track 2 was structured as a single phase challenge, running from January 27th, 2025 to May 12th, 2025. Given the substantial size and storage footprint of the generated single-cell RNA-seq datasets, synthetic datasets were not centrally collected, and no public leaderboard was implemented on the submission platform. Instead, the focus was placed on methodological transparency and reproducibility. Participants were required to submit:

- A short methodological description,
- Python source files implementing their methods
- Configuration file specifying hyperparameters, and
- An environment specification file to ensure computational reproducibility.

Rather than operating as a leaderboard-driven benchmark, Track 2 was positioned as an open research problem. Submitted approaches were evaluated and discussed through CAMDA extended abstract submissions.

## Logistics

**CAMDA extended abstract:** Participants of the both tracks were required to submit an accompanying CAMDA extended abstract by May 15, 2025 through the submission platform provided by the ISMB/ECCB 2025 conference (<https://www.iscb.org/ismbeccb2025/home>). Submissions were assessed using task-specific, multi-metric evaluation protocols designed to prevent over-optimization toward a single benchmark metric. The extended abstract was required to provide a detailed description of the proposed method, experimental design, and analytical findings, thereby enabling rigorous qualitative and quantitative assessment beyond leaderboard scores.

For Track 2 in particular, these abstracts carried increased weight due to the open-research format and the absence of a centralized leaderboard. As synthetic datasets were not collected centrally, methodological clarity, reproducibility documentation, and analytical justification presented in the abstract became central to the evaluation process.

**Github Starter Kit:** The participants were provided with a Github Starter Kit repository (<https://github.com/PMBio/Health-Privacy-Challenge> [^5^](https://sciwheel.com/work/citation?ids=19179093&pre=&suf=&sa=0) ) that contained baseline methods, evaluation metrics, and guidelines to prepare their submissions for respective tracks. They were asked to follow the template provided in the repository based Python to ensure reproducibility of their submission.

**Submission Platform:** The submissions were collected through the ELSA Benchmarking Platform [^3^](https://sciwheel.com/work/citation?ids=19179045&pre=&suf=&sa=0), where participants submitted the required files and thus, officially participated in the challenge.

REFERENCES

[1. Cancer Genome Atlas Research Network, Weinstein, J.N., Collisson, E.A., Mills, G.B., Shaw, K.R.M., Ozenberger, B.A., Ellrott, K., Shmulevich, I., Sander, C., and Stuart, J.M. (2013). The Cancer Genome Atlas Pan-Cancer analysis project. Nat. Genet. *45*, 1113–1120.](https://sciwheel.com/work/bibliography/48935)

[2. Yazar, S., Alquicira-Hernandez, J., Wing, K., Senabouth, A., Gordon, M.G., Andersen, S., Lu, Q., Rowson, A., Taylor, T.R.P., Clarke, L., et al. (2022). Single-cell eQTL mapping identifies cell type-specific genetic control of autoimmune disease. Science *376*, eabf3041.](https://sciwheel.com/work/bibliography/12772994)

[3. Health Privacy Challenge Organisation Team. ELSA Benchmarks Platform: Health  Privacy Challenge - CAMDA 2025.](https://sciwheel.com/work/bibliography/19179045) <https://benchmarks.elsa-ai.eu/?ch=4&com=introduction>, accessed: 2026-09-23

[4. Hilprecht, B., Härterich, M., and Bernau, D. (2019). Monte Carlo and Reconstruction Membership Inference Attacks against Generative Models. PoPETs *2019*, 232–249.](https://sciwheel.com/work/bibliography/11211853)

[5. Öztürk, H., Afonja, T., Jälkö, J., Binkyte, R., Rodriguez-Mier, P., Lobentanzer, S., Saez-Rodriguez, J., Fritz, M., Stegle, O., and Honkela, A. (2025). CAMDA Health Privacy Challenge 2025 Github Starter Kit for synthetic and private RNAseq generation (Github).](https://sciwheel.com/work/bibliography/19179093) <https://github.com/PMBio/Health-Privacy-Challenge>
