# Supplementary material for: Community benchmarking of generative models for privacy-preserving synthetic bulk RNA-seq data generation
Source: Genome Biol. 2026 Nov 9;27:355. doi: 10.1186/s13059-026-04326-1 (PMC13756914; doi:10.1186/s13059-026-04326-1)
Supplement: Supplementary file 2 [file 13059_2026_4326_MOESM2_ESM.docx]

Community benchmarking of generative models for privacy-preserving synthetic bulk RNA-seq data generation

Hakime Öztürk^*,1,2^, Tejumade Afonja^3^, Joonas Jälkö^4^, Ruta Binkyte^3^, Pablo Rodriguez-Mier^5^, Sebastian Lobentanzer^6^, Andrew Wicks^◇,7,8^, Jules Kreuer^◇,9^, Sofiane Ouaari^◇,9^, Nico Pfeifer^◇,9^, Shane Menzies^◇,10^, Sikha Pentyala^◇,10^, Daniil Filienko^◇,10^, Steven Golob^◇,10^, Patrick McKeever^◇,10^, Jineta Banerjee^◇,11^, Luca Foschini^◇,11^, Martine De Cock^◇,10,12^, Julio Saez-Rodriguez^5,13,14^, Mario Fritz^3^, Oliver Stegle^*,1,2^, Antti Honkela^*,4^

* : Corresponding authors ([hakime.oeztuerk@embl.de](mailto:hakime.oeztuerk@embl.de); [oliver.stegle@embl.de](mailto:oliver.stegle@embl.de); [antti.honkela@helsinki.fi](mailto:antti.honkela@helsinki.fi))

◇: Challenge participants that contributed method, synthetic data and CAMDA abstracts.

## Affiliations

**^1^European Molecular Biology Laboratory (EMBL), Genome Biology Unit, Heidelberg, Germany**

**^2^Division of Computational Genomics and Systems Genetics, German Cancer Research Center (DKFZ), Heidelberg, Germany**

**^3^CISPA Helmholtz Center for Information Security, Saarbrücken, Germany**

^4^**University of Helsinki, Helsinki, Finland**

^5^**Institute for Computational Biomedicine, Faculty of Medicine, Heidelberg University, Heidelberg, Germany**

^6^**Helmholtz Munich, Munich, Germany**

^7^**Division of Tumorigenesis and Molecular Cancer Prevention, German Cancer Research Center (DKFZ), Heidelberg, Germany**

^8^**DKFZ Hector Cancer Institute at the University Medical Center Mannheim, Mannheim, Germany**

^9^**Eberhard Karls Universität Tübingen, Tübingen, Germany**

^10^**University of Washington Tacoma, Washington, USA**

^11^**Sage Bionetworks, Seattle, USA**

^12^**Ghent University, Ghent, Belgium**

^13^**European Molecular Biology Laboratory, European Bioinformatics Institute (EMBL-EBI), Hinxton, UK**

^14^**Heidelberg University Hospital, Heidelberg, Germany**

# SUPPLEMENTARY TABLES

**Table S1:** **Complete list of evaluation metrics used to assess synthetic dataset quality.** Metrics are grouped into categories (e.g., utility, fidelity), with primary metrics highlighted for interpretation in the main manuscript and intermediate metrics providing additional diagnostic information reported in the supplementary material. Arrows indicate the desired optimization direction: upward (↑) for higher-is-better scores and downward (↓) for lower-is-better scores. Some of the metrics are computed on both training and held-out test partitions to evaluate generalization and detect potential overfitting. The public repository includes additional scripts for exploratory visualization and diagnostic analyses (e.g., PCA), which were not used in the reported results but are available for reproducibility and further exploration.

| **Category** | **Method Detail** | **Description** | **Role** | **Direction** |
| --- | --- | --- | --- | --- |
| Utility | Accuracy (Synthetic) | Downstream task performance for Train on Synthetic, Test on Real (TSTR) | Intermediate | **↑** |
| Utility | Accuracy (Real) | Downstream task performance for Train on Real, Test on Real (TRTR) | Intermediate | **↑** |
| Utility | Accuracy Relative | <https://saxarona.github.io/mathjax-viewer/?input=%20%5Ctext%7BAccuracy%7D_%7BTSTR%7D%20%2F%20%20%5Ctext%7BAccuracy%7D_%7BTRTR%7D%20%20#0> | Primary | **↑** |
| Utility | AUROC  (Synthetic) | TSTR | Intermediate | **↑** |
| Utility | AUROC  (Real) | TRTR | Intermediate | **↑** |
| Utility | AUROC Relative | <https://saxarona.github.io/mathjax-viewer/?input=%20%5Ctext%7BAUROC%7D_%7BTSTR%7D%20%2F%20%20%5Ctext%7BAUROC%7D_%7BTRTR%7D%20%20#0> | Primary | **↑** |
| Utility | PR Macro  (Synthetic) | TSTR | Intermediate | **↑** |
| Utility | PR Macro  (Real) | TRTR | Intermediate | **↑** |
| Utility | PR Macro Relative | <https://saxarona.github.io/mathjax-viewer/?input=%20%5Ctext%7BPR%20Macro%7D_%7BTSTR%7D%20%2F%20%20%5Ctext%7BPR%20Macro%7D_%7BTRTR%7D%20%20#0> | Primary | **↑** |
| Utility | F1 (Synthetic) | TSTR | Intermediate | **↑** |
| Utility | F1 (Real) | TRTR | Intermediate | **↑** |
| Utility | F1 Relative | <https://saxarona.github.io/mathjax-viewer/?input=%20%5Ctext%7BF1%7D_%7BTSTR%7D%20%2F%20%20%5Ctext%7BF1%7D_%7BTRTR%7D%20%20#0> | Primary | **↑** |
| Utility | Overlapping features % | Proportion of shared important features identified by TSTR and TRTR models | Primary | **↑** |
| Fidelity | Maximum Mean Discrepancy (MMD) (test) | Kernel-based distance between synthetic data and held-out real test data distributions | Primary | **↓** |
| Fidelity | Maximum Mean Discrepancy (MMD) (train) | Kernel-based distance between synthetic data and real training data distributions | Intermediate | **↓** |
| Fidelity | Kullback-Leibler (KL) divergence (test) | Divergence between synthetic and held-out real test data distributions | Primary | **↓** |
| Fidelity | Kullback-Leibler (KL) divergence (train) | Divergence between synthetic and real training data distributions | Intermediate | **↓** |
| Fidelity | Discriminative score F1 | F1 score for distinguishing synthetic and real dataset | Primary | **↓** |
| Fidelity | Distance-to-Closest | Average distance of synthetic dataset to the nearest real test data point | Intermediate | **↓** |
| Fidelity | Distance-to-Closest (baseline) | Average distance within real train dataset to the nearest test data point | Intermediate | **↓** |
| Fidelity | Distance-to-Closest Relative | Ratio of synthetic-to-real distances relative to the baseline, capturing fidelity normalized to intrinsic dataset structure, ideal value ≈ 1 | Primary | (~1) |
| Fidelity | Density | For each synthetic sample, the number of real-sample k-NN neighbourhoods (k=5) it falls within, normalized by k | Primary | (~1) |
| Fidelity | Coverage | Fraction of real samples whose k-NN neighbourhood (k=5) contains ≥1 synthetic sample | Primary | **↑** |
| Privacy | Area Under ROC Curve | The predictive performance of the membership inference attack (MIA) method | Primary | **↓** |
| Privacy | Area Under Precision Recall Curve | The predictive performance of the membership inference attack (MIA) method | Intermediate | **↓** |
| Privacy | TPR at FPR=0.1 | The ability of the attack to identify correct members when FPR is fixed at 10% | Primary | **↓** |
| Privacy | TPR at FPR=0.01 | The ability of the attack to identify correct members when FPR is fixed at 1% | Intermediate | **↓** |
| Biological Plausibility | Differential Expression (DE) True positive rate (TPR) | Agreement between synthetic and real datasets in detecting DE genes (e.g. between two subtypes) using Wilcoxon rank-sum test | Primary | **↑** |
| Biological Plausibility | DE False positive rate (FPR) | Rate of falsely detected DE genes in synthetic data compared to real data | Primary | **↓** |
| Biological Plausibility | Co-expression  TPR | Agreement between synthetic and real datasets in detecting co-expression network edges using hCoCena package | Primary | **↑** |
| Biological Plausibility | Co-expression  False edge rate | Number of falsely detected co-expression edges in synthetic data normalized by the number of edges in real network | Primary | **↓** |
| Biological Plausibility | Pathway  Mean KS | Two-sample Kolmogorov-Smirnov (KS) test between real/synthetic pathway score distributions (ssGSEA, 50 MSigDB Hallmark sets) | Primary | **↓** |
| Biological Plausibility | Pathway  Mean \|effect\| | Absolute standardized effect size (Cohen's d) computed as the mean difference (synthetic − real) divided by the pooled standard deviation. | Primary | **↓** |
| Biological Plausibility | Pathway  Var. ratio | Ratio of synthetic to real pathway-score variance (geometric mean across pathways) | Primary | (~1) |
| Biological Plausibility | Pathway  Frac. sig. (FDR) | Fraction of pathways with significant KS difference (Benhamini-Hochberg-FDR corrected across pathways) | Primary | **↓** |

**Table S2. Complete list of membership inference attacks applied in this study.** Seven attacks spanning three methodological categories (distance-based, density-based, and classifier-based) were evaluated across the TCGA-BRCA and TCGA-COMBINED datasets. Reference-anchored attacks (GAN-leaks calibrated, LOGAN-D1, DOMIAS-KDE) were restricted to TCGA-COMBINED, where a population-matched reference cohort (n=824) was available. For these attacks, four feature selection strategies were applied. All attacks were performed in a black-box, offline setting; full threat model assumptions are described in the Threat Model section in the main manuscript.

*FS*: Feature selection; *PCA*: Principal Component Analysis; *DVG*: Differentially Variable Genes; *SDEG*: Supervised Differentially Expressed Genes; *DG*: Discriminative Genes

| **Method** | **Approach** | **Reference-anchored** | **FS Applied** | **FS Methods** | **Dataset applied** | **Insights** |
| --- | --- | --- | --- | --- | --- | --- |
| **GAN-leaks** | Distance | No | No | - | BRCA,  COMBINED | Scores candidates by minimum distance to synthetic set; sensitive to outlier proximity rather than global distribution |
| **Monte Carlo (MC)** | Distance | No | No | - | BRCA,  COMBINED | Counts synthetic neighbours within adaptive threshold; sensitive to local density of synthetic samples |
| **GAN-leaks calibrated** | Distance (calibrated) | Yes | Yes | PCA, DVG, SDEG, DG | COMBINED | Calibrates distance scores against reference distribution; reduces false positives from population-level proximity |
| **DOMIAS-**  **KDE** | Density ratio | Yes | Yes | PCA, DVG, SDEG, DG | COMBINED | Estimates synthetic-to-reference density ratio via KDE; sensitive to regions where the generator oversamples training-like signals |
| **LOGAN-D1** | Classifier- based | Yes | Yes | PCA, DVG, SDEG, DG | COMBINED | Trains a neural-network discriminator to separate synthetic from reference samples; scores test points by the discriminator's synthetic-class output |
| **Confidence LR** | Classifier-based | No | No | - | BRCA,  COMBINED | Probes whether a logistic regression trained on synthetic data assigns higher confidence to training members |
| **Confidence RF** | Classifier-based | No | No | - | BRCA,  COMBINED | Same as above using Random Forest; captures non-linear decision boundaries |

**Table S3. Feature selection methods applied to reference-anchored membership inference attacks.** Four representations were evaluated to assess robustness of attack performance across feature spaces. All methods were fitted exclusively on the reference cohort and applied to synthetic and candidate test data, ensuring no membership information was incorporated into the feature extraction pipeline. Supervised method SDEG uses label information available in the synthetic release and represents an adversary with auxiliary biological knowledge.

| **Method** | **Type** | **Supervised** | **Dimension** | **Selection criteria** | **Insight** |
| --- | --- | --- | --- | --- | --- |
| **PCA** | Projection | No | 100 | Maximum variance | Captures global linear structure; not biology-aware but provides a standard baseline representation agnostic to gene identity |
| **Differentially Variable Genes (DVG)** | Feature selection | No | 100 | Variance ratio  (synthetic/  reference) | Retains genes where synthetic variance diverges most from reference; sensitive to distributional differences in expression variability |
| **Supervised Differentially Expressed Genes (SDEG)** | Feature selection | Yes | 100 | Mutual information with class labels (e.g. cancer subtype) | Selects genes most informative of cancer type/subtype in the synthetic data; reflects subtype-specific expression signal an adversary could exploit from a labelled release |
| **Discriminative Genes (DG)** | Feature selection | Yes | 100 | L1 Logistic Regression coefficient (synthetic vs reference) | Selects genes that maximally distinguish synthetic from reference samples; directly targets the feature space most likely to expose generator-specific biases |

**Table S4:** **Ablation analysis of input encoding strategies for the TCGA-BRCA dataset, comparing one-hot encoding and embedding-layer representations.** Fidelity and utility metrics are reported for both variants; “+” denotes embedding-based encoding and “−” denotes one-hot encoding.

| **Method** | **Fidelity** | | | | **Utility** | | | |
| --- | --- | --- | --- | --- | --- | --- | --- | --- |
|  | **MMD (test data) ↓** | **KL (test data) ↓** | **Discrm. Score ↓** | **Distance to closest ↓** | **Feature Overlap (%) ↑** | **AU-PR Relative ↑** | **AUROC Relative ↑** | **F1 Relative ↑** |
| **Real data** | 0.00 | 0.234 | - | 24.043 | - | 1.000 | 1.000 | 1.000 |
| **CVAE +** | 0.024 | 0.649 | 0.787 | 15.441 | 27.2 | 0.959 | 0.985 | 0.981 |
| **CVAE -** | 0.023 | 0.648 | 0.783 | 16.026 | 27.3 | 0.968 | 0.991 | 0.992 |
| **CVAE-GMM +** | 0.054 | 1.397 | 0.693 | 16.773 | 31.8 | 0.938 | 0.982 | 0.975 |
| **CVAE-GMM -** | 0.056 | 1.506 | 0.686 | 16.742 | 22.2 | 0.833 | 0.951 | 0.906 |
| **WGAN-GP+** | 0.033 | 0.830 | 0.705 | 19.361 | 13.7 | 0.918 | 0.967 | 0.938 |
| **WGAN-GP-** | 0.044 | 0.941 | 0.683 | 19.124 | 5.2 | 0.354 | 0.639 | 0.489 |

**Table S5:** **Ablation analysis of input encoding strategies for the TCGA-COMBINED dataset, comparing one-hot encoding and embedding-layer representations.** Fidelity and utility metrics are reported for both variants; “+” denotes embedding-based encoding and “−” denotes one-hot encoding.

| **Method** | **Fidelity** | | | | **Utility** | | | |
| --- | --- | --- | --- | --- | --- | --- | --- | --- |
|  | **MMD (test data) ↓** | **KL (test data) ↓** | **Discrm. Score ↓** | **Distance to closest ↓** | **Feature Overlap (%) ↑** | **AU-PR Relative ↑** | **AUROC Relative ↑** | **F1 Relative ↑** |
| **Real data** | -0.000 | 0.052 | - | 23.272 | - | 1.000 | 1.000 | 1.000 |
| **CVAE +** | 0.025 | 0.343 | 0.787 | 16.550 | 40.5 | 0.994 | 0.998 | 0.993 |
| **CVAE -** | 0.024 | 0.326 | 0.800 | 16.832 | 39.0 | 0.997 | 0.999 | 0.995 |
| **CVAE-GMM +** | 0.026 | 0.538 | 0.789 | 16.179 | 36.8 | 0.991 | 0.996 | 0.992 |
| **CVAE-GMM -** | 0.028 | 0.554 | 0.797 | 16.211 | 34.3 | 0.988 | 0.996 | 0.989 |
| **WGAN-GP+** | 0.014 | 0.158 | 0.953 | 20.130 | 24.0 | 0.987 | 0.997 | 0.983 |
| **WGAN-GP-** | 0.019 | 0.213 | 0.884 | 19.544 | 27.3 | 0.986 | 0.997 | 0.982 |

**Table S6:** **Resource consumption of the evaluated models during training and generation on the TCGA-BRCA dataset.** Training and generation were each performed for 871 samples. Columns report the hardware type and device name, wall-clock training and generation time (seconds), peak memory (GB), and total number of model parameters. Models were run on heterogeneous hardware (see Hardware columns); reported times and memory are therefore indicative of each model's requirements on its respective device and are not directly comparable across models.

| **Model** | **Hardware** | **Hardware Name** | **Training time (sec)** | **Generation time (sec)** | **Peak train memory (GB)** | **No.**  **params.** |
| --- | --- | --- | --- | --- | --- | --- |
| **CVAE** | GPU | NVIDIA GeForce RTX 3090 | ~116 | ~0.02 | 0.09 | 3.82M |
| **CVAE-GMM** | GPU | NVIDIA GeForce RTX 3090 | ~12 | ~0.58 | 0.08 | 3.45M |
| **Multivariate** | CPU | AMD EPYC 7601 32-Core Processor \| x86_64 | ~3e-06 | ~6.74 | 0.41 | - |
| **Embedded Diffusion** | GPU | NVIDIA GeForce RTX 5060 Ti | ~1081 | ~7.98 | 0.54 | 25.33M |
| **WGAN-GP** | GPU | NVIDIA GeForce RTX 3090 | ~39 | ~0.44 | 0.04 | 1.43M |
| **CTGAN** | GPU | NVIDIA GeForce RTX 3090 | ~154 | ~0.32 | 0.12 | 0.77M |
| **NMF** | CPU | Intel Core i7-8700B @ 3.20 GHz \| x86_64 | ~1.24 | ~0.24 | 0.23 | - |
| **DP-CVAE (ε=4)** | GPU | NVIDIA GeForce RTX 3090 | ~201 | ~0.03 | 1.76 | 3.82 |
| **DP-CTGAN (ε=50)** | GPU | NVIDIA GeForce RTX 3090 | ~1289 | ~6.20 | 0.19 | 0.77M |
| **P-MF** | CPU | Intel Core i7-8700B @ 3.20 GHz \| x86_64 | ~2.4 | ~0.60 | 0.22 | - |
| **DP-PGM (ε=4)** | CPU |  | ~9454 | ~4.40 | 1.08 | 0.02 |

**Table S7: Resource consumption of the evaluated models during training and generation on the COMBINED dataset.** Training and generation were each performed for 3,459 samples. Columns report the hardware type and device name, wall-clock training and generation time (seconds), peak memory (GB), and total number of model parameters. Models were run on heterogeneous hardware (see Hardware columns); reported times and memory are therefore indicative of each model's requirements on its respective device and are not directly comparable across models.

| **Model** | **Hardware** | **Hardware Name** | **Training time (sec)** | **Generation time (sec)** | **Peak memory (GB)** | **No.**  **params.** |
| --- | --- | --- | --- | --- | --- | --- |
| **CVAE** | GPU | NVIDIA GeForce RTX 3090 | ~60 | ~0.07 | 0.09 | 3.82M |
| **CVAE-GMM** | GPU | NVIDIA GeForce RTX 3090 | ~33 | ~1.92 | 0.08 | 3.44M |
| **Multivariate** | CPU | AMD EPYC 7601 32-Core Processor \| x86_64 | ~4e-06 | ~16.00 | 0.41 | - |
| **Embedded Diffusion** | GPU | NVIDIA GeForce RTX 5060 Ti | ~2560 | ~23.29 | 0.53 | 24.46M |
| **WGAN-GP** | GPU | NVIDIA GeForce RTX 3090 | ~157 | ~1.63 | 0.04 | 1.44M |
| **CTGAN** | GPU | NVIDIA GeForce RTX 3090 | ~260 | ~0.77 | ~0.12 | 0.79M |
| **NMF** | CPU | Intel Core i7-8700B @ 3.20 GHz \| x86_64 | ~4.42 | ~1.21 | ~0.37 | - |
| **DP-CVAE (ε=4)** | GPU | NVIDIA GeForce RTX 3090 | ~145 | ~0.12 | ~1.81 | 3.82M |
| **DP-CTGAN (ε=50)** | GPU | NVIDIA GeForce RTX 3090 | ~1491 | ~27.78 | ~0.20 | 0.79M |
| **P-MF** | CPU | Intel Core i7-8700B @ 3.20 GHz \| x86_64 | ~5.30 | ~1.50 | ~0.37 | - |
| **DP-PGM (ε=4)** | CPU | - | ~9601 | ~16.5 | ~1.13 | 0.04 |
