# Supplementary material for: Community benchmarking of generative models for privacy-preserving synthetic bulk RNA-seq data generation
Source: Genome Biol. 2026 Nov 9;27:355. doi: 10.1186/s13059-026-04326-1 (PMC13756914; doi:10.1186/s13059-026-04326-1)
Supplement: Supplementary file 3 [file 13059_2026_4326_MOESM3_ESM.docx]

Community benchmarking of generative models for privacy-preserving synthetic bulk RNA-seq data generation

Hakime Öztürk^*,1,2^, Tejumade Afonja^3^, Joonas Jälkö^4^, Ruta Binkyte^3^, Pablo Rodriguez-Mier^5^, Sebastian Lobentanzer^6^, Andrew Wicks^◇,7,8^, Jules Kreuer^◇,9^, Sofiane Ouaari^◇,9^, Nico Pfeifer^◇,9^, Shane Menzies^◇,10^, Sikha Pentyala^◇,10^, Daniil Filienko^◇,10^, Steven Golob^◇,10^, Patrick McKeever^◇,10^, Jineta Banerjee^◇,11^, Luca Foschini^◇,11^, Martine De Cock^◇,10,12^, Julio Saez-Rodriguez^5,13,14^, Mario Fritz^3^, Oliver Stegle^*,1,2^, Antti Honkela^*,4^

* : Corresponding authors ([hakime.oeztuerk@embl.de](mailto:hakime.oeztuerk@embl.de); [oliver.stegle@embl.de](mailto:oliver.stegle@embl.de); [antti.honkela@helsinki.fi](mailto:antti.honkela@helsinki.fi))

◇: Challenge participants that contributed method, synthetic data and CAMDA abstracts.

## Affiliations

**^1^European Molecular Biology Laboratory (EMBL), Genome Biology Unit, Heidelberg, Germany**

**^2^Division of Computational Genomics and Systems Genetics, German Cancer Research Center (DKFZ), Heidelberg, Germany**

**^3^CISPA Helmholtz Center for Information Security, Saarbrücken, Germany**

^4^**University of Helsinki, Helsinki, Finland**

^5^**Institute for Computational Biomedicine, Faculty of Medicine, Heidelberg University, Heidelberg, Germany**

^6^**Helmholtz Munich, Munich, Germany**

^7^**Division of Tumorigenesis and Molecular Cancer Prevention, German Cancer Research Center (DKFZ), Heidelberg, Germany**

^8^**DKFZ Hector Cancer Institute at the University Medical Center Mannheim, Mannheim, Germany**

^9^**Eberhard Karls Universität Tübingen, Tübingen, Germany**

^10^**University of Washington Tacoma, Washington, USA**

^11^**Sage Bionetworks, Seattle, USA**

^12^**Ghent University, Ghent, Belgium**

^13^**European Molecular Biology Laboratory, European Bioinformatics Institute (EMBL-EBI), Hinxton, UK**

^14^**Heidelberg University Hospital, Heidelberg, Germany**

## SUPPLEMENTARY FIGURES

**Fig. S1: Privacy–quality trade-off for differentially private models across privacy budgets (ε), on the TCGA-BRCA and TCGA-COMBINED datasets.** Each panel plots MIA risk under the strongest attack (AUC-ROC) against a quality metric (i.e. downstream utility (F1 relative) and differential-expression recovery TPR) with points coloured by DP model family and shaded by ε.

**Fig. S2: TGCA-BRCA** **Spearman correlation heatmap of the full set of fidelity and downstream utility metrics.** 13 metrics were included, and statistical significance was assessed using the Benjamini-Hochberg procedure controlling the false discovery rate (FDR) at α = 0.05. Significance levels are indicated as follows: *q < 0.05; ** q < 0.01; *** q < 0.001, where q represents the adjusted p-value.

**Fig. S3: TGCA-COMBINED** **Spearman correlation heatmap of complete fidelity and downstream utility metrics.** 13 metrics were included, and statistical significance was assessed using the Benjamini-Hochberg procedure controlling the false discovery rate (FDR) at α = 0.05. Significance levels are indicated as follows: *q < 0.05; ** q < 0.01; *** q < 0.001, where q represents the adjusted p-value.

**Fig. S4:** **Differential expression (DE) recovery difficulty across pairs in BRCA dataset.**  Differential expression (DE) recovery TPR across cancer type pairwise-comparisons with effect size ΔVST threshold set to 0.5 and FPR <=0.05. Each colored line represents a generative model. DP and non-DP methods are indicated with different symbols. The thick gray line indicates average (TPR) difficulty.

**Fig. S5:** **Differential expression (DE) recovery difficulty across pairs in COMBINED dataset.** TPR across cancer type pairwise-comparisons with effect size ΔVST threshold set to 0.5 and FPR <=0.05. Each colored line represents a generative model. DP and non-DP methods are indicated with different symbols. The thick gray line indicates average (TPR) difficulty.

**Fig. S6: Sensitivity of differential-expression (DE) recovery to the effect-size threshold ΔVST, across the TCGA-BRCA and TCGA-COMBINED datasets.** Recovery is evaluated at ΔVST = 0.0, 0.2, 0.5, and 1.0, shown as true positive rate (TPR) against realized false positive rate (FPR) in ROC-style panels (up- and down-regulation averaged). Increasing ΔVST imposes a stricter effect-size requirement; the FPR axis is restricted to ≤ 0.05 for visual clarity.

**Fig. S7: Pathway recovery in the TCGA-BRCA dataset, shown as signed effect sizes (Cohen's d) between synthetic and real ssGSEA scores across generative models and MSigDB Hallmark gene sets.** Positive values (red) indicate higher pathway activity in synthetic data, negative values (blue) lower activity; values near zero indicate faithful recovery.

**Fig. S8: Pathway recovery in the TCGA-COMBINED dataset, shown as signed effect sizes (Cohen's d) between synthetic and real ssGSEA scores across generative models and MSigDB Hallmark gene sets.** Positive values (red) indicate higher pathway activity in synthetic data, negative values (blue) lower activity; values near zero indicate faithful recovery.

**Fig. S9: TCGA-BRCA MIA TPR fixed at TPR = 0.1 performance across four MIA methods.** Membership inference risk was computed at each fold under five-fold cross-validation and the average performance is reported. The baseline performance is equal to 0.1.

**Fig. S10: TCGA-BRCA MIA AUC-ROC performance across four MIA methods.** Membership inference risk was computed at each fold under five-fold cross-validation and the average performance is reported. The baseline AUC-ROC corresponding to random guessing equals to 0.5.

**Fig. S11: TCGA-BRCA MIA AUC-ROC performance across four MIA methods.** Membership inference risk was computed at each fold under five-fold cross-validation and the average performance is reported. The baseline AUC-PR corresponding to random guessing equals to 0.8.

**Fig. S12: TCGA-COMBINED MIA TPR fixed at TPR = 0.1 performance across seven MIA methods.** Membership inference risk was computed at each fold under five-fold cross-validation and the average performance is reported. The baseline performance is equal to 0.1.

**Fig. S13: TCGA-COMBINED MIA AUC-ROC performance across seven MIA methods and four feature selection approaches.** Membership inference risk was computed at each fold under five-fold cross-validation and the average performance is reported. The baseline AUC-ROC corresponding to random guessing equals to 0.5.

**Fig. S14: TCGA-COMBINED MIA AUC-PR performance across seven MIA methods and four feature selection approaches.** Membership inference risk was computed at each fold under five-fold cross-validation and the average performance is reported. The baseline AUC-PR corresponding to random guessing equals to 0.8.

**Fig. S15: Impact of feature selection on membership-inference risk (TPR@FPR = 0.1) across the three reference-anchored attacks (GAN-leaks calibrated, DOMIAS, LOGAN), for the COMBINED dataset only.** Each panel shows one attack; rows are generative models and columns are feature-selection variants (PCA, DVG, SDEG, DG). Risk is computed per fold under five-fold cross-validation and averaged. The random-guessing baseline is TPR@FPR = 0.1.

**Fig. S16: Impact of feature selection on membership-inference risk (AUC-ROC) across the three reference-anchored attacks (GAN-leaks calibrated, DOMIAS, LOGAN), for the COMBINED dataset only.** Each panel shows one attack; rows are generative models and columns are feature-selection variants (PCA, DVG, SDEG, DG). Risk is computed per fold under five-fold cross-validation and averaged. The random-guessing baseline is AU-ROC= 0.5.

**Fig. S17: Agreement between feature-selection variants in how they rank generators by membership-inference risk, for the TCGA-COMBINED dataset only.** Each cell is the Spearman correlation of generator MIA risk rankings between two feature-selection variants, shown separately for each privacy metric (AUC-ROC, TPR@FPR = 0.1). High correlation (~ 1) indicates that the choice of feature selection does not reorder which generators are most vulnerable, whereas lower values indicate feature selection changes the ranking. Rankings are pooled as the (best-performing) maximum over the three reference-anchored attacks; n = 11 generators.

**Fig. S18: Spearman correlations between privacy measures across generative models, per MIA attack.** For each attack, generator-level membership-inference risk under the two privacy metrics (AUC-ROC and TPR@FPR = 0.1) and the raw nearest-neighbour distance (distance-to-closest-record) are correlated across generators. Each row/point is an attack and correlations are shown for each metric pair. Correlations are computed across n = 11 generators (fold-averaged); values are descriptive (uncorrected p).

**Fig. S19: TGCA-BRCA** **Spearman correlation heatmap of selected metric from four evaluation axes: fidelity, downstream utility, biological plausibility and privacy.** 11 metrics were included, and statistical significance was assessed using the Benjamini–Hochberg procedure controlling the false discovery rate (FDR) at<https://www.codecogs.com/eqnedit.php?latex=%5Calpha%20%3D%200.05#0>. Significance levels are indicated as follows: *q < 0.05; ** q < 0.01; *** q < 0.001, where q represents the adjusted p-value.

**Fig. S20: TGCA-COMBINED** **Spearman correlation heatmap of selected metric from four evaluation axes: fidelity, downstream utility, biological plausibility and privacy.** 11 metrics were included, and statistical significance was assessed using the Benjamini–Hochberg procedure controlling the false discovery rate (FDR) at<https://www.codecogs.com/eqnedit.php?latex=%5Calpha%20%3D%200.05#0>. Significance levels are indicated as follows: *q < 0.05; ** q < 0.01; *** q < 0.001, where q represents the adjusted p-value.

**Fig. S21: Noise–quality trade-off for the MVN baseline across additive Gaussian noise levels, on the TCGA-BRCA and TCGA-COMBINED datasets**. Each panel plots MIA risk under the strongest attack (AUC-ROC) against a quality metric (i.e. downstream utility (F1 relative) and differential-expression recovery TPR), with points colored by noise level (n).

**Fig. S22: Privacy–quality trade-off for the P-NMF model across privacy budgets (ε), on the TCGA-BRCA and TCGA-COMBINED datasets.** Each panel plots MIA risk under the strongest attack (AUC-ROC) against a quality metric: downstream utility (relative F1) and differential-expression recovery (TPR). P-NMF is not formally differentially private: its calibrated noise (parameterized by ε) applies only to the released per-cluster gene means, not to the synthetic data, therefore, ε here bounds the privacy of those released statistics rather than providing a data-level DP guarantee.
